# Disrupted hippocampal replay is associated with reduced offline map stabilisation in an Alzheimer’s mouse model

**DOI:** 10.1016/j.cub.2025.12.061

**Published:** 2026-01-29

**Authors:** Sarah Shipley, Marco P. Abrate, Robin Hayman, Dennis Chan, Caswell Barry

**Affiliations:** 1 Research Department of Cell and Developmental Biology, UCL, Gower St, London WC1E 6BT, UK; 2 MRC Cognition and Brain Sciences Unit, University of Cambridge, 15 Chaucer Rd, Cambridge CB2 7EF, UK; 3 Institute of Neurology, UCL, Queen Square, London WC1N 3BG, UK; 4 Institute of Cognitive Neuroscience, UCL, Alexandra House, 17-19 Queen Square, London WC1N 3AZ, UK

## Abstract

Alzheimer’s disease is characterised by progressive memory decline associated with hippocampal degeneration. However, the specific physiological mechanisms underlying hippocampal dysfunction in the disease remains poorly understood - improved knowledge may aid diagnosis and identify new avenues for therapeutic intervention. We investigated how disruptions in hippocampal reactivations relate to place cell stability and spatial memory deficits in an Alzheimer’s mouse model. Using the App knock-in mouse model ‘NL-G-F’, we conducted simultaneous behavioural and electrophysiological recordings in a radial arm maze. NL-G-F mice exhibited significant impairments in memory performance, demonstrated by an increased propensity to revisit arms, compared to wild-type controls. These memory deficits were associated with reduced stability of hippocampal place cells, particularly pronounced following rest periods. Crucially, while wild-type mice showed enhanced place cell stability after quiescence, NL-G-F mice failed to exhibit this consolidation. Although the rate of hippocampal reactivation events during rest remained unchanged, analysis of replay content revealed significantly degraded replay quality in NL-G-F mice. This degradation manifested as disrupted cell recruitment and reduced co-firing structure within reactivation events, both of which predicted the failure of offline place cell stabilisation. Together, these findings suggest that compromised reactivation quality may underlie disruptions in offline consolidation processes, offering a potential mechanism for memory dysfunction in Alzheimer’s disease.

## Introduction

The hippocampus plays a central role in memory for places and events, spatial navigation, and system-level memory consolidation^1–4^. A key functional basis of the hippocampal role in navigation and spatial memory is the stable, spatially selective activity of place cells, primarily found in hippocampal regions CA3 and CA1^5,6^. The population activity of these cells is considered a core element of a cognitive map, providing a representation of self-location necessary to support flexible navigation to remembered goals^7^. During quiescence, place cells engage in transient bursts of activity, reactivating temporally compressed trajectories through the environment (replay). These reactivation events, often synchronised with cortical activity^8,9^, are widely considered a central mechanism supporting consolidation^10–12^. Within the hippocampus, replay events modulate synaptic efficacy, stabilising place fields^13,14^. Experimental disruption of sharp-wave ripples, which often accompany reactivations, leads to place field destabilisation, underscoring the role of offline processes in maintaining spatial representations^10,11^.

This understanding of the role of reactivation in memory consolidation has implications for understanding the pathophysiology underlying Alzheimer’s disease (AD). While memory impairment is the hallmark symptom of clinical AD, the pathological cascade begins decades earlier during an extended preclinical phase characterised by amyloid-β accumulation without significant neurodegeneration^15^. During this preclinical stage, amyloid pathology triggers aberrant neural network activity, particularly hippocampal hyperactivation, which precedes and may contribute to the eventual neuronal loss seen in clinical disease^16,17^. This hyperactivation correlates with subtle cognitive changes detectable through sensitive testing, even while gross memory function remains intact.

Understanding these early network disruptions is crucial for two reasons. First, the advent of anti-amyloid immunotherapies as the first wave of new therapies with the potential to modify disease and delay progression to dementia has increased the importance of early disease detection, given disease-modifying treatments are of greatest benefit in the earliest stages of AD when the extent of irreversible neurodegeneration is lowest^18^. In this context, greater understanding of the physiological mechanisms underlying the earliest cognitive changes in AD will benefit efforts to deliver tests with superior ability to detect the disease in its initial stages. However, identification of hippocampal pathophysiological changes would have benefits beyond early diagnosis alone, in that it would provide a mechanistic target against which to direct future symptom-targeted interventions.

Given that amyloid pathology precedes other AD markers and accumulates early in hippocampal regions^19,20^, examining hippocampal dysfunction in amyloid models provides insight into the earliest pathological stages of the disease. Modern knock-in models of amyloid pathology offer particular advantages for investigating these preclinical mechanisms, as they recapitulate amyloid accumulation without the confounding APP overexpression artefacts seen in earlier transgenic models. These models allow us to examine how initial amyloid pathology disrupts hippocampal network function—including place cell stability and offline consolidation—during the critical window before widespread neurodegeneration obscures the primary effects of amyloid on neural circuits.

Building upon our understanding of hippocampal function and its relevance to AD, studies in rodent AD models have reported a range of abnormalities in place cell firing characteristics. These include changes to spatial information, field size, and firing rate, which precede changes in other brain structures^21–25^. While there is some variability in results arising from different mouse models, reflecting differences in the expression of AD molecular pathology and associated neuronal loss^23,26,27^ the most consistent finding across studies is the deterioration of place cell stability over various time-frames, both between and within recording sessions^21,24–27^.

Given that hippocampal reactivations are critically involved in maintaining place field stability^14,28^, disrupted reactivation sequences may contribute to the reduction in field stability seen in AD. Earlier studies have identified a decrease in the quantity or power of sharp-wave ripples in several rodent AD models^26,29–36^. However, these local field potential features provide only an indirect measure of reactivation events. Their detection relies on power in specific frequency bands, making them vulnerable to the oscillatory changes that occur in mouse models of AD pathologies^32,37–39^, and crucially, they cannot reveal the content or quality of the replayed information. More recent work by Prince et al. has begun to address these limitations by examining cellular-level reactivations, identifying altered recruitment patterns in an amyloid mouse model^40^. Nevertheless, the relationship between reactivation content—the information actually replayed—and its impact on place cell stability remains largely unexplored. Furthermore, existing findings require validation in modern knock-in models, which offer more physiologically relevant protein expression compared to overexpression models^41,42^. Most critically, whether degraded reactivation content prevents the offline consolidation of spatial representations—and how this relates to memory impairment—has not been established.

Here we investigated reactivations in rest periods (‘offline’) as a mechanism underlying place field instability and subsequent memory impairment in an amyloid mouse model of AD. We examined place cell stability across different timescales while mice performed a familiar 8-arm radial maze task, simultaneously assessing two distinct forms of memory performance–within trial preference for unique arms as opposed to arm revisits (analogous to working memory), and preference for rewarded arms (analogous to reference memory). To ensure clinical relevance, we employed an App knock-in model (App^NL-G-F^), circumventing the problems associated with APP overexpression seen in many amyloid models of AD^41,43^. To directly assess hippocampal reactivations, we identified epochs of multi-unit activity (MUA) from single cell recordings in CA1 during rest periods^44–48^. We found that while wild-type mice showed enhanced place cell stability following rest, App^NL-G-F^ mice failed to exhibit this consolidation. This failure of rest-dependent stabilisation was accompanied by impaired memory performance on the radial maze, particularly the inability to avoid previously visited arms within trials.

Analysis of replay content—rather than the rate at which sharp-wave ripples occurred—revealed that while the number of reactivation events remained unchanged (contrary to previous sharp-wave ripple-based studies^26,32,35^), replay quality was significantly degraded in App^NL-G-F^ mice. This degradation, characterised by disrupted cell recruitment and diminished co-firing structure, predicted the failure of place field consolidation. Our findings point to a possible mechanistic link from disrupted reactivation quality through place cell instability to memory impairment in AD, suggesting that the fidelity of reactivation patterns, rather than their frequency, could serve as both an early biomarker of hippocampal dysfunction and a potential therapeutic target.

## Results

### NL-G-F mice show a reduced preference for unvisited arms in a radial arm maze task

**Figure 1:**
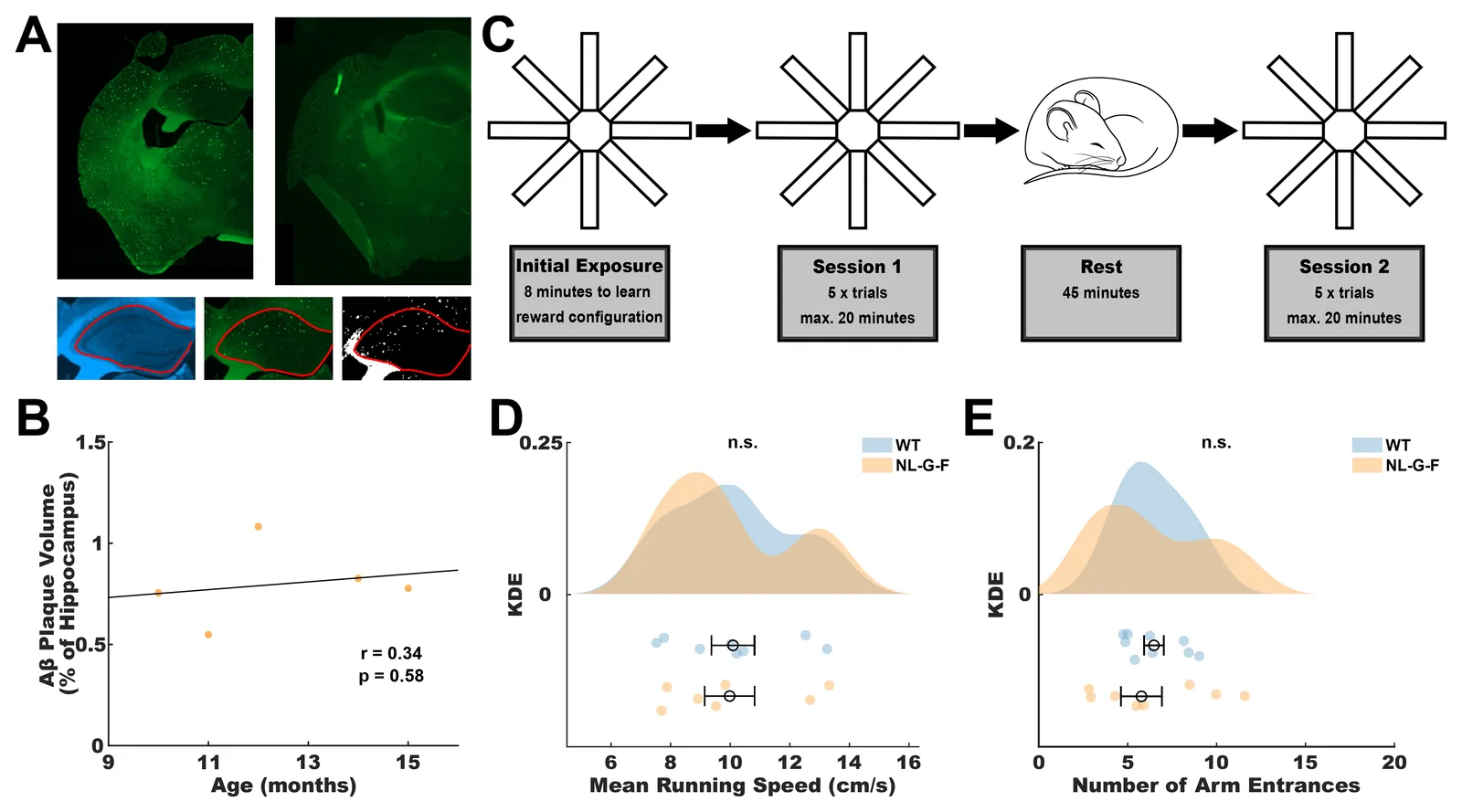
NL-G-F mice show amyloid pathology but similar running behaviour to WT on the radial arm maze **A**. Amyloid plaques were present in NL-G-F (top left) but not WT (top right) hippocampus, visualised with ThioflavinS staining. Bottom row: DAPI staining was used to define the hippocampal ROI (left, red line), which was applied to the ThioflavinS image (centre) and binarised using an Otsu filter (right) for quantification (see Methods). **B**. Plaque volume did not correlate with age in NL-G-F mice (n_NL-G-F_=5). **C**. Daily experimental procedure on the radial arm maze, which consisted of 8 arms (80cm x10cm) connected by an octagon (10cm sides). **D**. Mean running speed and **E**. number of arm entrances per trial was not different for WT and NL-G-F mice. Bars show mean and SEM, shaded area shows Kernel Density Estimate (KDE). *n*_*WT*_=9 mice, *n*_*NL-G-F*_=8 mice. n.s.= not significant in two-sample t-test.

Eight male App^NL-G-F^/Chat-cre mice (from here on referred to as ‘NL-G-F’) and nine age matched wild-type littermate controls (Figure 1A-B; two-sample t-test: M_WT_=12.22 months, Range_WT_=8,17; M_NL-G-F_ =11.50 months, Range_NL-G-F_=7,15; t=0.50, p=0.63. Pearson’s correlation between plaque volume and age: r(3)=0.34, p=0.577) were trained to run on an 8-arm radial arm maze until they were consistently running to the arm ends to receive rewards and achieving good coverage of the maze (Figure S1). During training, all arms were rewarded. Concurrently, extracellular electrodes (64 channels per mouse) were gradually lowered until they reached the CA1 cell layer (neural recordings performed in 15 of the mice; 8 wild-type and 7 NL-G-F). Once competency had been achieved and the cell layer reached, mice progressed to the experimental recordings (Figure 1C, Methods). Therefore, for all mice at the start of testing the maze was a familiar environment but the task was novel.

Mice completed a total of 3-5 test days (Table S1). On each test day, four of the arms were randomly selected as reward arms and baited. The reward configuration remained consistent throughout all 10 trials in a test day but changed across days. In each trial, mice were placed onto the central platform of the maze and allowed to forage for rewards. They were removed from the maze after 4 minutes or after all four rewards were consumed, whichever came first (Figure 1C). Running behaviour was evaluated based on two parameters: mean running speed and the number of arm entries per trial. No significant differences were observed between NL-G-F mice and wild-type controls in either running speed (Figure 1D; two-sample t-test: M_WT_=10.09 cm/s, M_NL-G-F_=9.98 cm/s, t=0.10, p=0.92) or number of arm entries (Figure 1E; two-sample t-test: M_WT_=6.48, M_NL-G-F_= 6.44, t=0.03, p= 0.97).

To complete the task efficiently, mice should enter only the reward arms and avoid revisiting any arm, thus minimising travel distance and time. We assessed memory performance using two key metrics: the preference for visiting reward arms and the preference for visiting unique, previously unvisited, arms. For each of these, we developed a metric scored between 0 and 1, expressing animals’ preference for reward or unvisited arms whilst controlling for the total number of arm entrances (see Methods). The score represents the probability that an animal choosing the same number of arms at random would make a greater number of errors than observed. These scores allow us to quantify the animals’ ability to remember both the location of rewards and their previous choices, independent of their overall activity level. These metrics were analysed across 10 trials within each day, and across different days using linear mixed-effects models which represent the effect of a predictor variable on outcome and accounted for individual variability among the mice.

**Figure 2:**
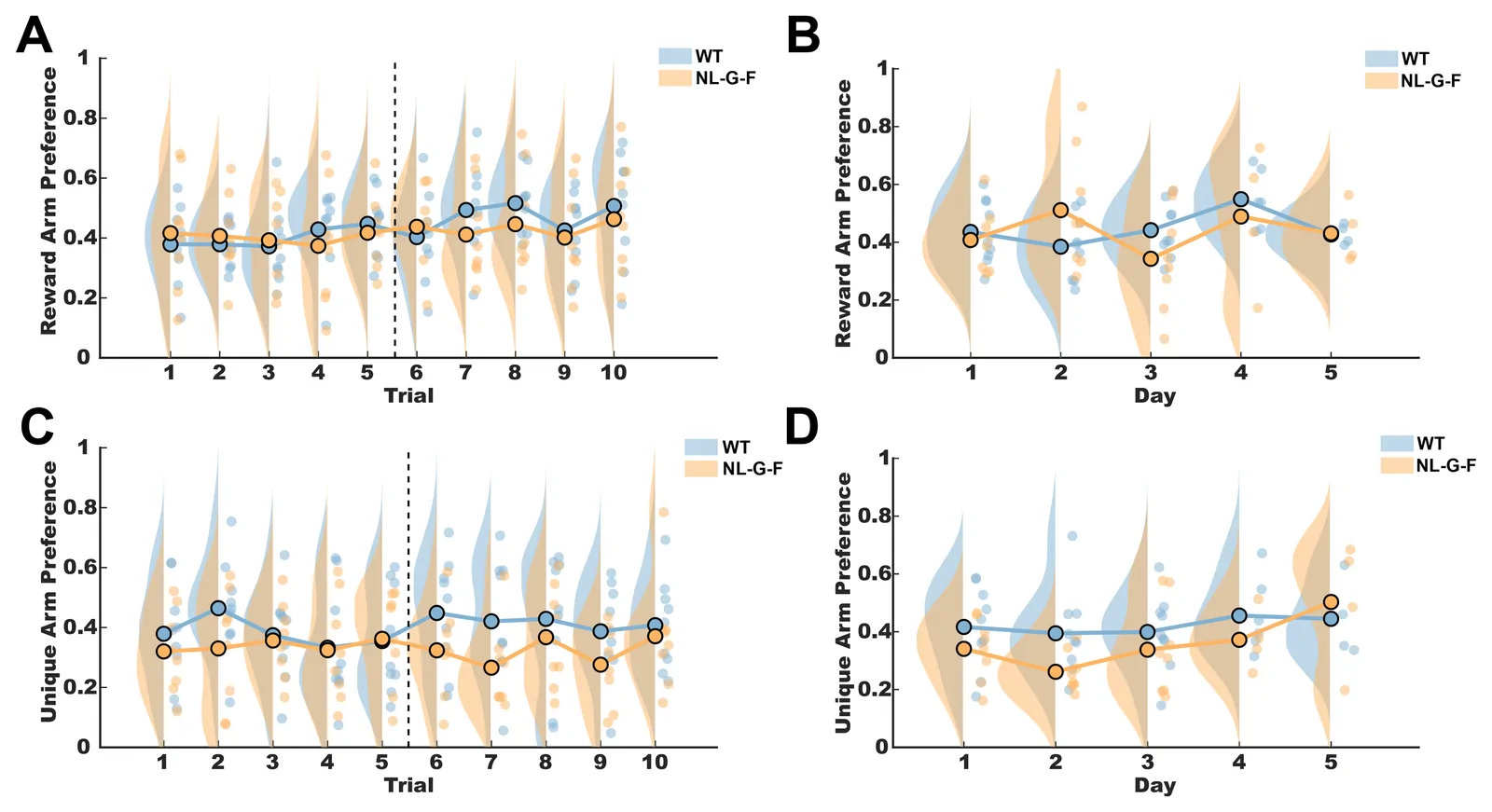
NL-G-F mice have a reduced preference for unvisited arms in an 8-arm radial arm maze task. **A-B**. Mean and KDE of reward arm preference scores across trials **(A)** and days **(B)**. There was an overall increase in reward arm preference across trials (p<0.01) but no other significant factors. Dashed line represents the position of the rest recording separating sessions 1 and 2. **C-D**. Same as **A-B** but for unique arm preference score. NL-G-F mice showed a decreased preference for visiting unique arms (p<0.05), and the preference decreased across trials (p<0.05) and days (p<0.01), though positive interaction effects existed between trial and day (p<0.01), and day and genotype (p<0.05). **A-D** significance tested using LME models (Performance Score ~ 1 + Trial*Day + Trial*Genotype + Day*Genotype + Trial:Day:Genotype + (1 | *Mouse))*. **A,C**. *n*_*WT*_=9 mice, *n*_*NL-G-F*_=8 mice. **B,D**. Days 1-3: *n*_*WT*_=9, *n*_*NL-G-F*_=8. Day 4: *n*_*WT*_=6, *n*_*NL-G-F*_=4. Day 5: *n*_*WT*_=4, *n*_*NL-G-F*_=4. Related to Table.S2.

Both wild-type and NL-G-F mice demonstrated an increase in their preference for reward arms over the course of the 10 trials within a day (Figure 2A-B, Table S2; Estimate=0.026, SE=0.010, t(675)=2.667, p <0.01), indicating that they were capable of learning the reward configuration. However, there was no effect of genotype and interaction effects were not significant (Genotype: Estimate=0.188, SE=0.101, t(675)=1.869, p=0.062; Day: Estimate=0.034, SE=0.021, t(675)=1.636, p=0.102; Trial x Day: Estimate=-0.004, SE=0.003, t(675)=-1.078, p=0.281; Trial x Genotype: Estimate=-0.025, SE=0.014, t(675)=-1.729, p=0.084; Day x Genotype: Estimate=-0.052, SE=0.031, t(675)=-1.697, p=0.090).

In contrast to wild-type animals, NL-G-F mice were more likely to return to arms they had already visited (Figure 2C-D; Estimate=-0.243, SE=0.105, t(675)=-2.315, p=0.021). We also saw significant main effects of Trial and Day, with unique arm preference decreasing over time and trials (Trial: Estimate=-0.024, SE=0.010, t(675)=-2.413, p=0.016; Day: Estimate=-0.060, SE=0.021, t(675)=-2.892, p=0.004). Equally, significant interactions existed between trial and day (Estimate=0.01, SE=0.003, t(675)=3.02, p=0.002) and day and genotype (Estimate=0.070, SE=0.030, t(675)=2.296, p=0.022), but not trial and genotype (Estimate=0.017, SE=0.014, t(675)=1.177, p=0.240). Collectively, these results demonstrate that NL-G-F mice specifically failed to avoid revisiting arms. Although wild-type mice showed an increased likelihood to revisit arms across trials and days, this was less pronounced in NL-G-F mice, as their score improved relative to wild-types.

### Deficits in place cell stability predict memory performance in NL-G-F mice

**Figure 3:**
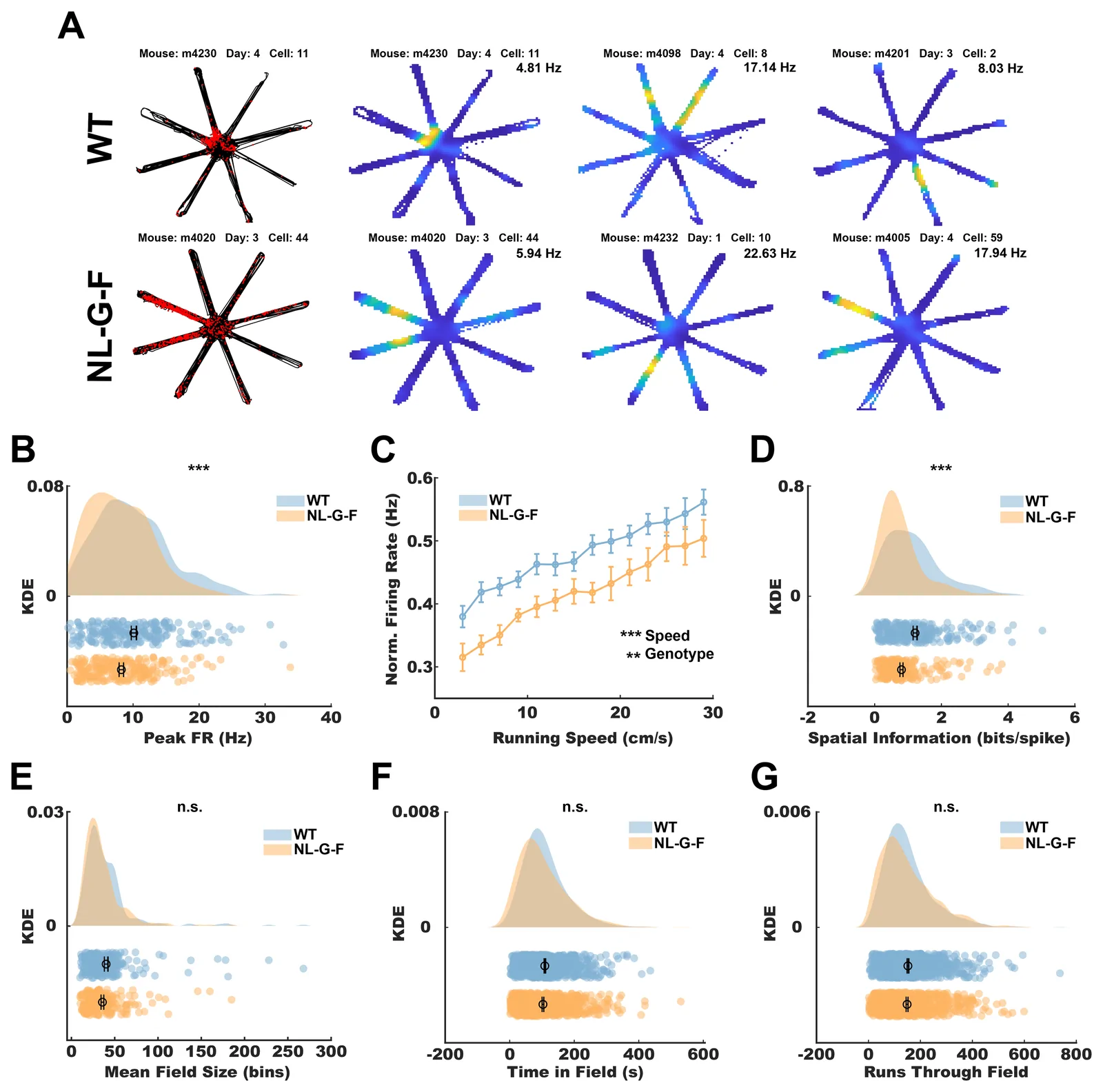
Firing rate of place cells is deteriorated in NL-G-F mice but fields preserved. **A**. Spike raster plots (left) and ratemaps (right) of example place cells recorded in WT (top) and NL-G-F (bottom) mice. Rasters represent individual spike positions recorded over one day (concatenated across 10 trials). Ratemaps are colour coded from 0 to peak firing rate, labelled above each map. The first ratemap corresponds to the raster plot. **B**. Peak firing rate of place cells was decreased in NL-G-F mice (t-test, p<0.001). **C**. Firing rate increased with speed of running within the firing field (p<0.001). Despite the overall decrease in firing in NL-G-F mice (p=0.0019), there was no interaction effect (p=0.32). Firing rate was normalised by the peak firing rate of the cell. LME model: Firing Rate ~ 1 + Geno*Speed + (1 | *mouse) + (1* | *day). n*_*WT*_=965 cell days, *n*_*NL-G-F*_=806 cell days. **D**. Spatial Information of place cells was diminished in NL-G-F mice (t-test, p<0.001). **E**. Mean field area, averaged across all fields recorded from single cells, was not different between WT and NL-G-F mice (t-test, p=0.1). **B,D,E**. Cells recorded on the first test day across all mice (*n*_*WT*_=232, *n*_*NL-G-F*_=222). **F**. WT and NL-G-F mice spent similar amounts of time within individual cell’s firing fields (p=0.08). **G**. Mice also made a similar number of runs through fields (p=0.51). Scatter plots show individual cell-days (n_WT_=965, n_NL-G-F_=806). Bars show mean and SEM. Related to Figure S1,S2 and Table.S1,S2.

Place cells were recorded from mice navigating the radial arm maze (Figure 3A, S1, Table S1; total across days: n_WT_=965, n_NL-G-F_=806), with key firing properties assessed only on the first test day to avoid repeated inclusion of cells (n_WT_=232, n_NL-G-F_=222; Figure S2). NL-G-F mice exhibited a reduced peak firing rate compared to controls (Figure 3B; two-sample t-test: M_WT_=10.11Hz, M_NL-G-F_=8.22Hz, t=3.66, p<0.001). When normalised by peak firing rate to control for differences in overall activity, the positive relationship between running speed and firing rate within fields was similar across genotypes (Figure 3C; running speed: Estimate= 0.006, SE=0.0005, t(696)=11.42, p<0.0001; running speed x genotype interaction: Estimate=0.007, SE=0.0007, t(696)=0.99, p=0.32) yet there was a main effect of genotype (Estimate=-0.08, SE=0.02, t(696)=-3.11, p=0.001) indicating that cells are less active both in absolute terms and relative to their own dynamic range. Lower spatial information content of spikes (Figure 3D; two-sample t-test: M_WT_=1.21 bits/spike, M_NL-G-F_=0.80 bits/spike, t=5.42, p<0.0001), was similarly indicative of impaired spatial encoding. However, no significant difference in firing field size was observed between the genotypes (Figure 3E; two-sample t-test: M_WT_=40.11 bins, M_NL-G-F_=35.82 bins, t=1.63, p=0.103).

**Figure 4:**
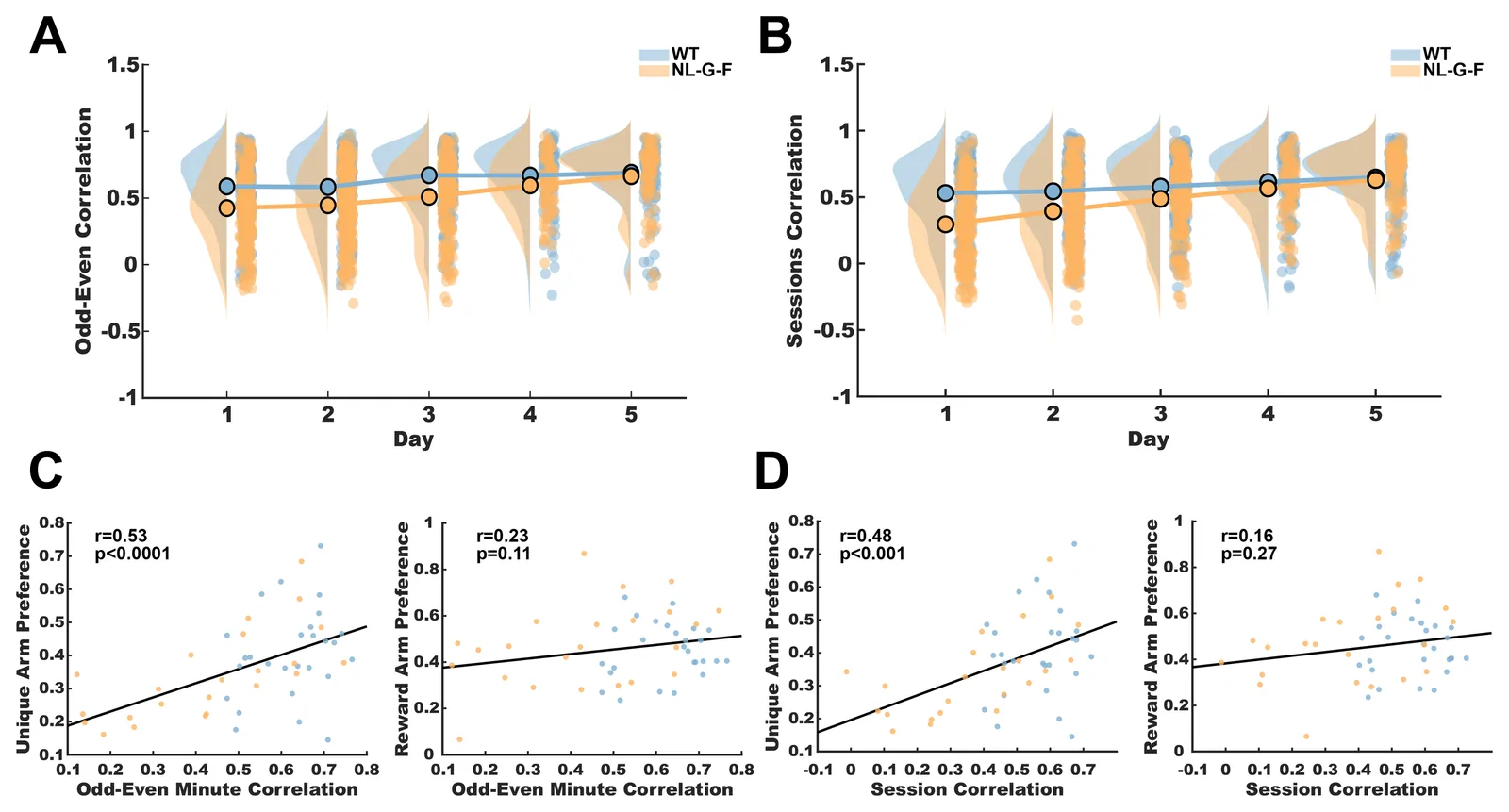
Reduced place cell stability in NL-G-F mice corresponds to a decreased preference for unvisited arms. **A**. Odd-even minute rate map correlations were significantly lower in NL-G-F mice (p<0.001) and increased across days (p<0.01). **B**. Session-to-session (session 1 vs. 2) correlations of rate maps were also reduced in NL-G-F mice (p<0.0001) and showed an increase over days (p < 0.001), with NL-G-F mice improving more over time than WT mice (p<0.05). **A-B**. Pearson correlations of paired rate maps, excluding unvisited spatial bins. Statistical analysis was conducted with an LME model: Correlation value ~ 1 + Geno*Day + (1 | *Mouse). n*_*WT*_=965 cell days, *n*_*NL-G-F*_=806 cell days. **C**. Place cell stability across odd and even minutes was significantly correlated with unique arm preference (left) but not reward arm preference (right). **D**. Place cell stability across sessions was also significantly correlated with unique arm preference (left) but not reward arm preference (right). **C-D**. Line represents Spearman correlation. Each dot represents a recording day for a single mouse (*n*=50 days; colour coded by genotype for visualisation: *n*_*WT*_*=27, n*_*NL-G-F*_=23). Related to Figure S3 and Table.S2.

Place cell stability was evaluated using spatial correlations between ratemaps corresponding to two different time horizons: those composed of odd versus even minutes within a session, and those composed of the first versus second recording sessions of each day. Across session stability, reflecting longer time periods, was strongly reduced in NL-G-F mice (Figure 4B; Estimate=-0.28, SE=0.07, t(1765)=-4.25, p<0.0001), and improved across days (Estimate=0.02, SE=0.007, t(1765)=3.44, p<0.001). Notably, a significant interaction between genotype and day was observed, suggesting that while the stability of NL-G-F place fields was initially poor, it improved across test days (Estimate=0.02, SE=0.01, t(1765)=2.39, p<0.05). To test whether behavioural sampling contributed to this stability deficit, we compared the number of visits to place fields and time spent in fields across genotypes and found no differences (Figure 3F-G, two-sample t-tests: time in field: M_WT_=108.7s, M_NL-G-F_= 103.04s, t(1752)=1.70, p=0.08; runs through field: M_WT_=152s, M_NL-G-F_=148s, t(1752)=0.67, p=0.51). A linear mixed-effects model revealed opposing relationships between experience of fields and stability (time in field: Estimate = 0.004, t(1746) = 12.7, p<0.001; runs through field: Estimate = -0.002, t(1746) = -11.89, p<0.001) however there were no interactions with genotype (Time in field x genotype: Estimate = -0.0008, t(1746)=-1.91, p=0.05. Runs through field x genotype: Estimate =0.0003, t(1746) =1.14, p=0.25). The main effect of genotype remained significant (Estimate = –0.14, t(1746) = –2.87, p < 0.01), indicating that reduced stability in NL-G-F mice is not explained by differences in the animals’ patterns of place field visitation.

Odd-even minute correlations were also lower in NL-G-F mice, reflecting reduced stability relative to wild-types over shorter time periods (Figure 4A; Estimate=-0.23, SE=0.06, t(1767)=-3.57, p<0.001). We also saw a primary effect of day, indicating that odd-even minute stability increased in both groups (Estimate=0.02, SE=0.007, t(1767)=3.12, p<0.01) - with no interaction between genotype and day (Estimate=0.007, SE=0.01, t(1767)=0.75, p=0.45).

Both stability measures showed a strong positive association with a preference for unique arms (Figure 4C-D; Odd-Even Correlation: r(50)=0.55, p<0.0001; Session Correlation: r(50)=0.48, p<0.001) - indicating that animals with more stable place fields were less inclined to revisit arms. Due to the high stability in the wild-type group, this effect was driven by the greater variability in the stability of place fields in NL-G-F mice (Figure S3). Conversely, no such relationship was observed with reward arm preference (Figure 4C-D; Odd-Even Correlation: r(50)=0.19, p=0.20; Session Correlation: r(50)=0.16, p=0.27). These findings highlight a specific link between the stability of hippocampal place cell representations and the ability to avoid revisiting arms within a given trial.

### Impaired place cell stability and offline consolidation in NL-G-F mice

**Figure 5:**
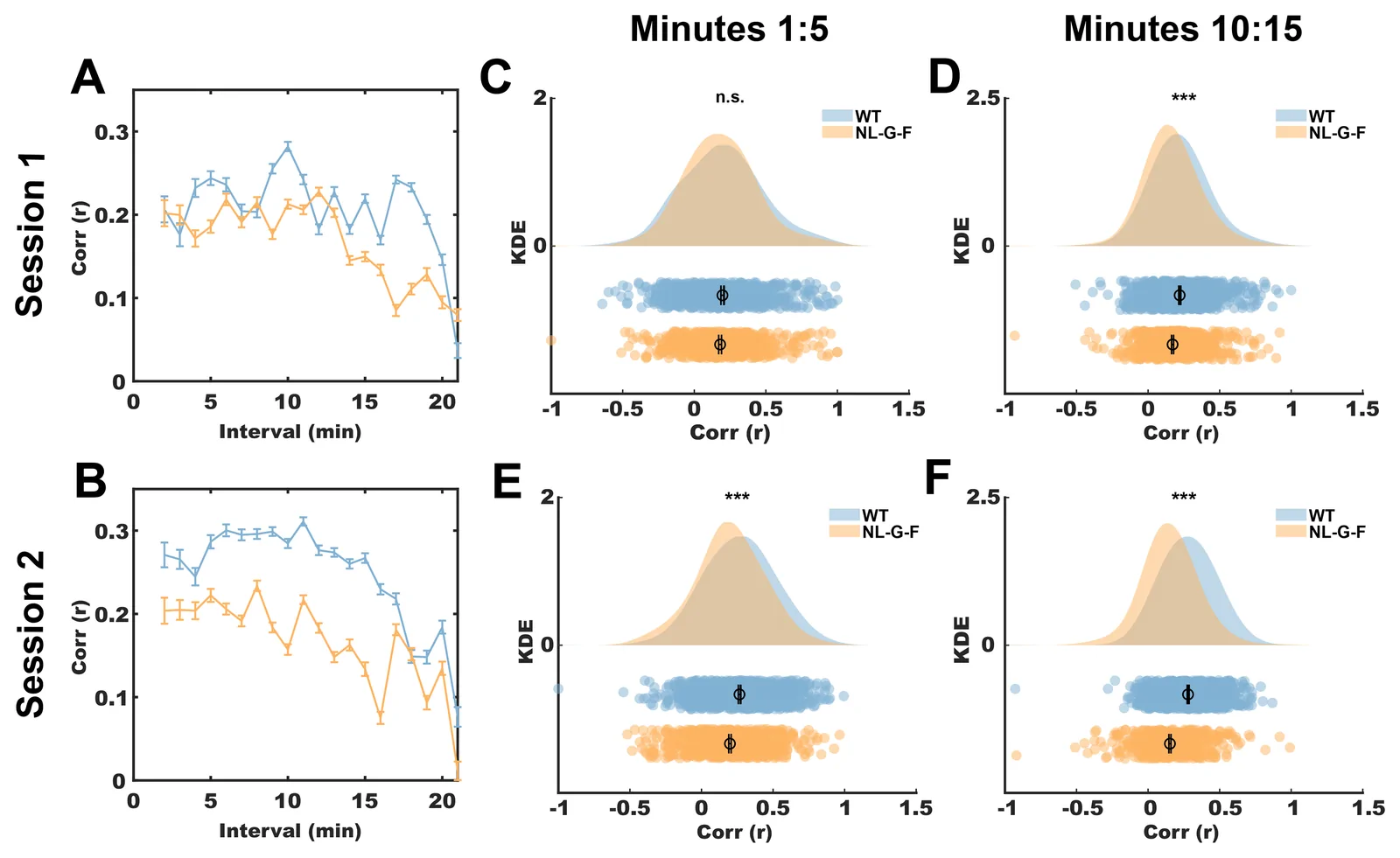
Place cell stability degrades more rapidly and does not increase during rest in NL-G-F mice. **A,B**. Pearson correlations (±SEM) between minute-by-minute ratemaps at increasing temporal lags within session 1 (A) and session 2 (B). Both temporal lag and NL-G-F genotype were associated with decreased correlation (p<0.0001 and p=0.001, respectively). The genotype × session interaction (p<0.0001) reveals that WT mice showed increased stability after rest while NL-G-F mice did not. The temporal lag × genotype interaction (p<0.0001) indicates differential rates of stability decay between genotypes. LME model: Correlation ~ 1 + Session×Genotype + Lag×Genotype + (1|Mouse). Only overlapping spatial bins were analysed (n_WT_= 305,688, n_NL-G-F_=228,781 ratemap pairs). **C**. At short temporal delays (1-5 minutes) in session 1, correlation values between WT and NL-G-F mice were not significantly different (*n*_*WT*_=690, *n*_*NL-G-F*_=663 ratemap pairs; p=0.27). **D**. At longer temporal delays (10-15 minutes) in session 1, NL-G-F mice exhibited decreased ratemap correlations compared to WT mice (*n*_*WT*_=947, *n*_*NL-G-F*_=740 ratemap pairs; p<0.0001). **E**. In session 2, following a rest session, NL-G-F mice had lower ratemap correlations at short temporal delays (*n*_*WT*_=949, *n*_*NL-G-F*_=791 ratemap pairs; p<0.01). **F**. Decreased ratemap correlations in session 2 were exacerbated over longer temporal delays (10-15 minutes; *n*_*WT*_=942, *n*_*NL-G-F*_=677 ratemap pairs; p<0.0001). **C-F**. p-values from 2-sample t-tests. Plots show individual Pearson correlation values and KDE. Bars show mean and SEM. Related to Figure S3 and Table.S2.

To investigate temporal variation in place cell stability between NL-G-F and wild-type mice, we generated minute-by-minute ratemaps throughout each session and calculated correlations between all possible pairs at different temporal lags (Figure 5A-B). For example, we correlated ratemaps separated by 1 minute, 2 minutes, and so on, up to the maximum lag possible within a session. These correlations were then assessed through linear mixed-effects modelling (see Methods), allowing us to examine how place cell stability degraded as a function of temporal lag and how this degradation differed between genotypes.

Consistent with our previous analyses, overall, correlations were lower in NL-G-F mice (Figure 5A-B; NL-G-F Estimate=-0.098, SE=0.003, t(295980)=-3.24, p=0.001), corresponding to a general reduction in the place cell stability in NL-G-F animals. We also saw two additional main effects, a negative influence of temporal delay (Estimate=-0.005, SE=0.0003, t(295980)=-16.27, p<0.0001) and a positive effect of session (Estimate=0.04, SE=0.03, t(295980)=16.60, p<0.0001). Indicating, as expected, that place cell responses became increasingly dissimilar over more distant time points and that spatial responses tended to be more stable in the second session of the day.

Notably, we found significant interactions between session and genotype (Estimate=-0.03, SE=0.004, t(295980)=-5.77, p<0.0001), indicating that wild-type mice showed enhanced place cell stability following rest whereas NL-G-F animals did not, suggesting a failure of offline consolidation. When these results were extended to investigate change across days, an improvement across days was seen at a slower rate in the NL-G-F group (Figure S3; Day x Genotype interaction, Estimate:-0.004, t(295980)=-2.32, p= 0.02), further indicating a failure of map stabilisation. To examine these temporal dynamics in detail, we grouped correlations into 5-minute blocks and compared stability over short (<5 minutes; Figure 5C,E) and long (10-15 minutes; Figure 5D,F) temporal delays. These intervals were chosen to maximise data inclusion while accounting for instances where some mice completed trials in under four minutes. During session 1, the stability of ratemaps separated by short temporal delays (<5 minutes) was not different between genotypes (M_WT_=0.20, M_NL-G-F_=0.18 r-value; two-sample t-test, t(1351)=1.11, p=0.27). However, ratemaps separated by 10 to 15 minutes were less stable in NL-G-F mice compared to wild-type mice (M_WT_=0.22, M_NL-G-F_=0.17 r-value; two-sample t-test, t(1685)=5.14, p<0.0001). This indicates that ratemaps in wild-type mice were stable over longer intervals. In session 2, following a 45 minute rest, NL-G-F place fields were less stable than wild-type cells even at short temporal delays (<5 minutes; M_WT_=0.27, M_NL-G-F_=0.20 r-value; two-sample t-test, t(1738)=5.70, p<0.0001), with the size of the effect increasing over longer temporal delays (M_WT_=0.28, M_NL-G-F_=0.15 r-value; two-sample t-test, t(1617)=13.50, p<0.0001). These results suggest that, following rest, place fields from wild-type mice had higher initial levels of stability, and that stability was maintained over longer intervals.

Collectively, these findings indicate a general reduction in place cell stability across longer temporal lags within a session, an effect which was more prominent in NL-G-F mice. Furthermore, place fields in wild-type animals became more stable after the rest period between sessions, while NL-G-F mice failed to show this improvement. We therefore hypothesised that variations in offline reactivations between genotypes might contribute to the observed disparities in ratemap stability.

### Coherent offline reactivations stabilise the hippocampal map

The stabilisation of place fields has been attributed to the reactivation of place cells during sleep ^14,28^, suggesting a potential mechanism for the observed disparity between wild-type and NL-G-F mice. To explore this, we identified epochs of elevated multi-unit activity (MUA), which has previously been validated as a method of identifying reactivation events^8,44–47^, and assessed differences in decoded replay trajectories, event frequency, and cell recruitment patterns between genotypes.

**Figure 6:**
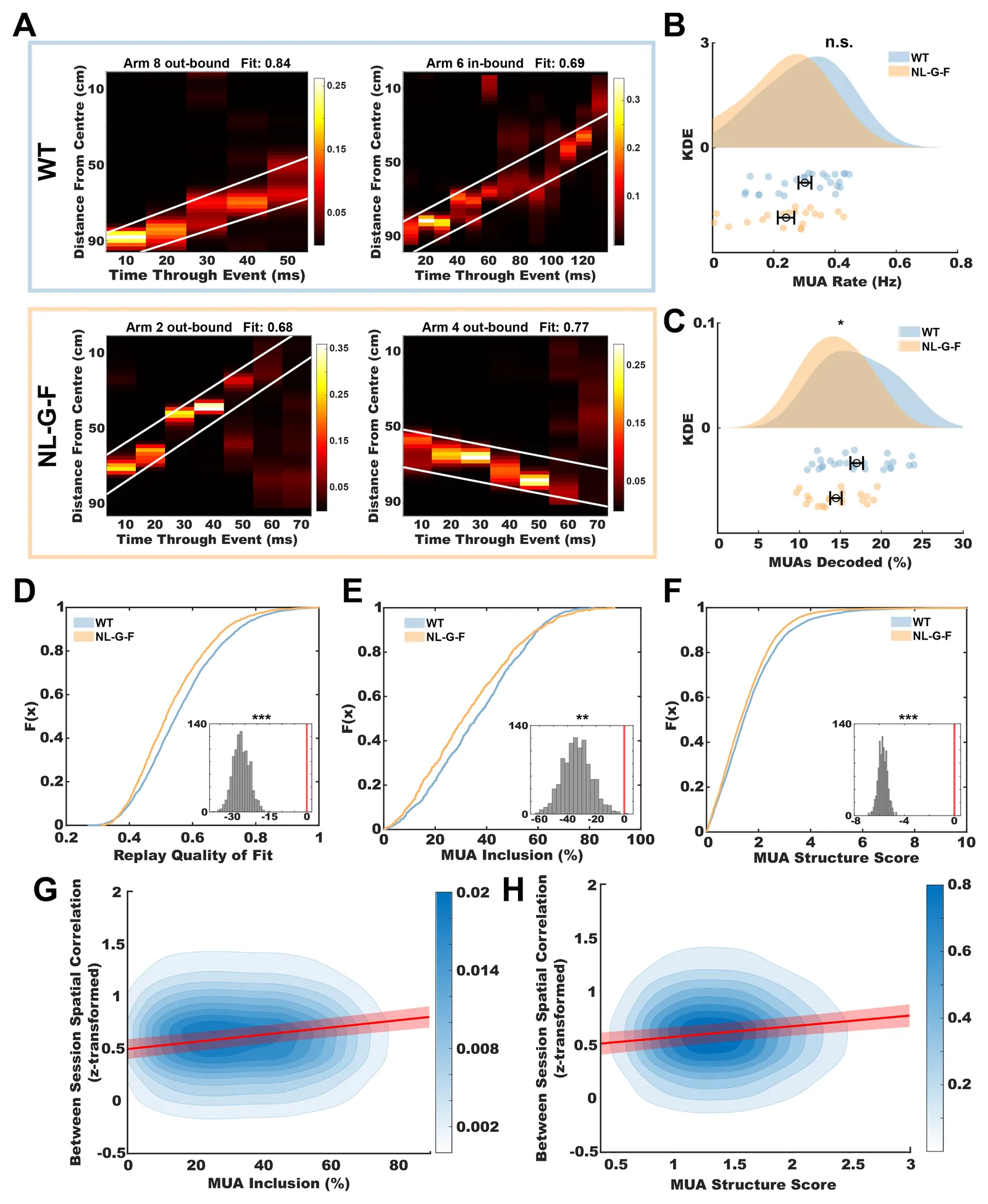
Cell recruitment in reactivations contributes to instability in place cell firing. **A**. Example replay events from WT (top, blue box) and NL-G-F (bottom, orange box) mice. Plots show the posterior-probability matrix with the best-fit trajectory (white lines). **B**. MUA rate in the rest recording was not different between WT and NL-G-F mice (p= 0.07, *n*_*WT*_=27, *n*_*NL-G-F*_=20 mouse-days). **C**. The percentage of all MUAs that contained a replay trajectory that passed the shuffle threshold was higher in WT than NL-G-F mice (p<0.05, *n*_*WT*_=27, *n*_*NL-G-F*_=20 mouse-days). **D**. Quality of fit for decoded replay trajectories was higher in WT than NL-G-F mice (n_WT_=3989, n_NL-G-F_=2222; p<0.001). **E**. The percentage of MUAs to which each cell was recruited was reduced in NL-G-F mice (*n*_*WT*_=965, *n*_*NL-G-F*_=806 cell days; p<0.01). **F**. The level of structure within MUAs, measured as the likelihood of cell pair co-activity under a null hypothesis of statistical independence, was also reduced in NL-G-F mice (*n*_*WT*_=48465, *n*_*NL-G-* *F*_=21921 cell pairs; p<0.001). **D-F**. Difference in area under the curve (AUC) was tested for significance using a bootstrapping procedure (1000 iterations), with the resulting distribution (inset) being compared to zero (red line). **G-H**. Linear mixed effects model demonstrated that recruitment to MUAs **(G)** and structure in MUAs **(H)** were both predictive of cell stability across sessions (z-transform of the Pearson correlation of ratemaps; *n*=1519 cell days; both p<0.0001). Red line represents the LME model estimate. Blue area shows the KDE contour plot of individual cells; z-transform was performed for the intercept of each mouse-day. LME model: *z-Scored Session Corr ~ 1 + [MUA structure OR MUA inclusion] + (1* | *Mouse:Day)*. Related to Figure S4,S5 and Table.S2.

The frequency at which MUA events occurred did not differ between wild-type and NL-G-F mice (Figure 6B; two-sample t-test: M_WT_=0.30Hz, M_NL-G-F_=0.24Hz, t(45)=1.83, p= 0.07) and did not change across days (Figure S5). Similarly we found no difference in the brain states during the rest recording (Figure S4) or the rate of ripple detection between genotypes (Figure S5), in line with previous reports by Brady *et al*.^30^. To assess replay content within MUAs, we decoded the maze positions represented by active place cells using a Bayesian approach^8,44,47,49^ (Figure 6A). We first identified which arm of the maze was represented in each MUA event by calculating posterior probability distributions across all spatial bins using the ensemble place cell activity, then summing these probabilities separately for each arm. Given that place cells exhibit strong directional selectivity on linear tracks ^50^, we treated inbound and outbound trajectories as distinct, effectively analysing 16 possible trajectories (8 arms × 2 directions). The arm and direction with the highest summed probability was selected for further analysis. Next, we decoded the detailed spatial trajectory within the selected arm by calculating the posterior probability matrix for positions along that arm across time. We then fit linear trajectories to this matrix, testing all possible speeds (Figure S5) and starting positions to find the trajectory that captured the greatest probability mass (see Methods). To validate replay trajectories, we compared their goodness-of-fit against two shuffle controls: a cell-identity shuffle (spike trains randomly reassigned between cells, preserving temporal structure) and a column-rotation shuffle (posterior probability matrix columns independently rotated)^44,51,52^. Trajectories were considered valid only if they exceeded both shuffle thresholds. While both genotypes showed replay events, NL-G-F mice had significantly fewer events that passed these validation criteria. Only 14.45% of MUAs in NL-G-F mice contained replay trajectories that exceeded shuffle thresholds, compared to 16.99% in wild-type controls (Figure 6C, S5; two-sample t-test: t(45)=2.34, p=0.02). Of those that did meet this threshold, the replay quality - the proportion of the posterior probability matrix accounted for by the best-fit trajectory - was also reduced in the NL-G-F mice (Figure 6D; M_WT_= 0.56, M_NL-G-F_= 0.53, bootstrapped differences in AUC: p<0.0001).

These replay deficits in NL-G-F mice suggest reactivations are impaired, however decoding techniques are fallible to changes in place cell representations on the maze - including place field stability. To avoid such confounds, we looked at the recruitment of individual cells to MUA events and observed a difference in how individual neurons participated - place cells in NL-G-F mice being recruited to a lower proportion of events than in wild-type mice. (Figure 6E; M_WT_=36.06%, M_NL-G-F_=33.06%, bootstrapped differences in AUC: p=0.001). Thus, to assess how coordinated populations of cells were recruited to MUAs, we developed a structure score that measures whether pairs of cells co-activate differently than expected by chance. Specifically, we compared the observed frequency of cell pairs firing together within MUAs to the expected frequency if each cell fired independently (see Methods). This yields an absolute z-score where zero indicates random co-activation, while higher values indicate non-random patterns—either consistent co-firing or consistent avoidance of co-firing - independent of the rate at which individual cells were recruited. MUA structure in wild-type animals was greater than in NL-G-F mice (Figure 6F; M_WT_=1.52, M_NL-G-F_=1.39, bootstrapped differences in AUC: p<0.0001), indicating that wild-type MUAs contained more organised cell assemblies with consistent firing relationships, likely conveying more coherent spatial information.

We therefore examined whether these reactivation deficits were associated with the reduced place cell stability observed in NL-G-F mice. Using linear mixed-effects models (see Methods), we tested whether reactivation measures predicted place cell stability across sessions, accounting for repeated measurements within mice and recording days. Both MUA structure and cell recruitment significantly predicted cross-session place cell stability (Figure 6G,H; MUA Structure: Estimate=0.10, SE=0.022, t(1517)=4.53, p<0.0001; MUA Inclusion: Estimate=0.003, SE=0.0005, t(1767)=6.80, p<0.0001). These findings indicate that coordinated cell recruitment and structured co-firing within reactivations support the maintenance of stable spatial representations across rest periods. The disruption of these processes in NL-G-F mice thus suggests a mechanistic link between aberrant reactivations and impaired spatial memory.

## Discussion

In this study, we employed a multifaceted approach combining behavioural assessment in a radial arm maze task with simultaneous single-cell extracellular recordings to investigate hippocampal function, including reactivations, in the NL-G-F mouse model of Alzheimer’s disease (AD). We identified a strong association between place cell stability and memory performance, both of which were impaired in NL-G-F animals. Although this correlation does not establish direct causality and was driven primarily by variability within the NL-G-F group—as wild-type mice exhibited uniformly high stability—the within-genotype correlation suggests that the degree of spatial instability scales with behavioural impairment. Moreover, the similarity in field sampling across genotypes (Figure 3) indicates that behavioural differences do not account for the observed instability, supporting a mechanistic relationship between place cell stability and task performance. Notably, we observed a time-dependent deterioration of place cell stability. Over short time frames, place cells in NL-G-F mice were stable comparable to those in wild-type animals. However, over longer periods, the stability of place fields in NL-G-F mice was markedly reduced relative to controls. This deficit was particularly pronounced following rest periods, after which the stability of place fields in wild-type animals increased whereas in NL-G-F mice it did not, resulting in a widening disparity between the two groups over longer temporal delays within sessions. To investigate the mechanisms underlying these differences, we examined multi-unit reactivations during rest periods. We found that place cells were recruited to fewer multi-unit activity (MUA) events in NL-G-F mice, and that those MUAs were less structured, with cells reactivating in less consistent combinations. Linear modelling revealed that both MUA recruitment and MUA structure were predictors of across-session stability, indicating that coordinated reactivations are linked to place cell stability and may contribute to maintaining the spatial map.

Understanding the neural mechanisms underlying memory impairment has major implications for AD management, not just in terms of yielding outcomes that may aid early diagnosis or track response to treatment but also in potentially identifying a target for future interventions aiming to restore normal physiological function. As such, a key aim of this study was to investigate the link between neural changes and memory performance. Using a radial arm maze task, we were able to evaluate different facets of memory, revealing a specific deficit in the preference for unique arms, rather than reward arms. This finding aligns with previous research that has shown reduced performance in alternating Y-mazes^41^ and Morris-water-maze tasks^53^ in the same NL-G-F mouse model, though other studies have reported no such deficits^54^. While the neural distinctions between these types of memory remain unclear, our findings suggest that the observed deficits are linked to the varying timescales required for learning different aspects of the task. In this study, preference for unique arms is an intra-trial measure of memory, relying on rapid, one-shot learning of ‘just visited’ but familiar locations. In contrast, preference for reward arms involves repeated learning across multiple trials within a day, showing improvement with practice. Consistent with this distinction, following quiescence, the stability of spatial representations in NL-G-F mice—as measured at both intra-trial and inter-session lags—was initially reduced. Indeed, this reduction in place cell representation stability was explicitly correlated with a decreased preference for visiting unique arms. Conversely, across repeated days of exposure, these same stability measures gradually improved, though at a reduced rate for intra-trial stability, whilst inter-session stability of place representations in NL-G-F animals recovered towards normal levels. Broadly this suggests that NL-G-F mice retain sufficiently normal neural dynamics to benefit from cumulative learning across multiple exposures to an environment, which may explain the relative preservation of reference memory observed by us and other researchers in amyloid mouse models of AD^55^. This sensitivity to different learning timescales—and the capacity for improvement across days—may contribute to our understanding of the variable outcomes reported across memory tasks that differ in cognitive demands and experimental design^54,56,57^. Together, these findings underscore the importance of offline reactivation processes in supporting the incremental consolidation of spatial representations over extended periods.

Notably, the most pronounced difference between the two genotypes was in the stability of place cells after the 45-minute rest period - place cells in wild-type animals increased in stability, whereas those in NL-G-F mice did not. Hippocampal replay - compressed reactivations of hippocampal sequences^12,58,59^ - typically occur during quiescence and are known to play a central role in maintaining place cell stability^11,14,28,46^. Prior work has seen deficits in the occurrence of sharp-wave ripples - transient LFP phenomena that often co-occur with reactivations - in App transgenic mouse models of AD^33,35^, tau models^26,29,36^ and an APOE4 knock-in model^32^. However this is far from a consistent finding across studies, likely because accurate measurement is confounded by differences in the oscillatory frequency of sharp-wave ripples between AD and wild-type animals^60–63^.

Multi-unit activity (MUAs), the method used here, provides a direct measure of hippocampal pyramidal cell activity and offers a more reliable indicator of reactivation than sharp-wave ripples used in previous studies, which—although no difference was found here—may be sensitive to changes in oscillatory dynamics^47,64^. Importantly, single unit recordings enabled us to directly assess the content of reactivations - to our knowledge, the first decoding of replay trajectories in an AD model. Using this measure, we found no difference in the rate at which MUAs occurred between AD and wild-type animals. However, we observed that the replay content was degraded - with fewer events containing identifiable replay trajectories, and a marked decrease in the quality of decoding even within those that passed the detection threshold. Furthermore, the more robust measures of cell recruitment, which avoid the impact of pathology-driven changes in cell firing, demonstrated that both the ability to recruit place cells into MUAs and to do so in a consistent structure of cell co-firings was reduced in NL-G-F animals. These findings complement work by Prince et al.^40^, who identified impaired cell recruitment and co-ordination within reactivations in an APP overexpression model of AD. However, we extend these results by demonstrating that degraded reactivation quality predicts the failure of offline place field stabilisation, suggesting a plausible mechanistic pathway from aberrant replay to spatial memory deficits.

This triad of findings—impaired spatial memory, reduced place cell stability, and disrupted reactivation structure— suggests that coordinated cell recruitment during MUAs is closely linked to spatial map stabilisation during rest, which may support subsequent short-term spatial memory performance. The functional deficit in this amyloid mouse model of Alzheimer’s disease lies not in the ability of hippocampal networks to generate reactivations but in their precise ordering and internal structure. It follows that this aberrant activity is insufficient to engage the normal plastic processes required to stabilise the activity of place cells.

Further work is needed to fully clarify the causes of disrupted MUA structure in Alzheimer’s disease models. Prior work in both this and other rodent models of AD has identified changes in gamma and theta-band oscillations^32,37–39^, which may be relevant given recent findings showing a causal link between theta-phase place cell firing and subsequent replay occurrence^65^. This suggests that alterations in neural oscillations during active behaviour in early AD could contribute to the reactivation deficits we observed during rest. Establishing this complete pathway—from oscillatory disruption through reactivation failure to behavioural impairment—would provide valuable mechanistic insights into AD-related memory deficits and reveal potential targets for symptom-specific therapeutic interventions.

## Resource Availability

### Materials Availability

This study did not generate new unique reagents.

## Star methods

### Experimental Model and Study Participant Details

All mice were from the App^NL-G-F^/ChAT-cre strain, generated by crossing the App^NL-G-F^ Alzheimer’s model (Saito et al., 2014) with ChAT-IRES-Cre (B6;129S6-Chattm2(cre)Lowl/J; Jackson Laboratory, Stock no: 006410). This model expresses a humanised form of the App gene carrying mutations that lead to amyloidosis and synaptic loss, while maintaining expression levels comparable to the endogenous murine App gene. The colony was maintained with breeding pairs homozygous for ChAT-IRES-Cre and heterozygous for App^NL-G-F^. App^NL-G-F^ homozygotes comprised the experimental group, while wild-type littermates served as controls. Electrophysiological recordings were performed on 15 male mice: 8 wild-type and 7 App^NL-G-F^, with two additional mice (one per genotype) completing behavioral testing only.

Mice were aged to a minimum of 7 months prior to implantation (M_WT_=12.25 months, range_WT_=8,16; mean_NL-G-F_=11.43, range_NL-G-F_=7,15; t_(15)=_0.73, p=0.476),ensuring plaque presence throughout the brain^41^ (Figure1).They were housed communally under 12-hour reversed light-dark cycles until surgery, then individually housed and food-restricted to 90% free-feeding weight (or 35g if obese). All procedures followed institutional (University College London) and national ethical standards under the UK Animals (Scientific Procedures) Act 1986.

## Method Details

### Surgery

Mice were implanted with a single microdrive carrying 64 electrodes twisted into 16 tetrodes. Tetrodes were formed from 12 μm polyimide-coated nickel-chrome wire (California Fine Wire Co.) and platinum-plated to reduce impedances below 150kΩ. Microdrives consisted of a single body (Axona Ltd.) mounted to a screw allowing simultaneous advancement of all tetrodes.

Following stereotaxic placement, a midline incision exposed the skull for three craniotomies: one for a grounding screw and two for stabilization screws. A trephine drill created a craniotomy above left hippocampus for microdrive implantation (coordinates: 2.0mm posterior to bregma, 1.8mm lateral to midline, 0.8mm dorsal from brain surface). A protective metal sheath was lowered over exposed tetrodes and the surgical site secured with dental cement fixing the microdrive to skull.

### Place Cell Screening

Following one-week post-surgical recovery, mice were handled and habituated to the experimental room. The drive was connected to the recording system and mice foraged freely in an open-field environment (80cm diameter) for 20-40 minutes until sufficient environmental coverage was achieved. Neural data were reviewed for hippocampal activity signatures (theta during running, sharp-wave ripples, pyramidal cell waveforms and activity bursts) and clustered spikes were reviewed for identification of place fields. The number of spatially-tuned cells was assessed visually, with animals progressing to experimentation upon identifying sufficient spatial cells (minimum 20) or when spatial cell numbers consistently decreased. If insufficient spatial cells were identified, tetrodes were advanced by turning the microdrive screw

### Behavioural Training

Experiments used a radial eight-arm maze with 80cm x 10cm arms connected by an octagonal centre platform (24.7cm diameter, 10cm sides). Each arm terminated in a reward well with raised edges preventing distant visibility, plus a second well behind each arm filled to obscure odour cues.

Following one-week post-surgical recovery, mice were habituated to the setup. Initial exposure allowed untethered exploration, followed by tethered eight-minute foraging sessions with two-minute inter-trial intervals in a holding box while the maze was cleaned and wells replenished. The holding box was a modified plastic flower-pot with removed bottom and side-split for containment without handling. Mice were held in the maze centre with randomly rotated release positions to prevent route learning.

During habituation, rewards were provided on all arms to encourage exploration, with uneaten rewards removed to discourage revisits. Habituation lasted 2-7 days, ending when animals consistently ran to multiple arm ends and regularly consumed rewards. Mice then progressed to the test phase.

### Behavioural Task

On each test day, four randomly selected arms contained rewards, with constraints that at least two arms differed from the previous day and reward arms were not all consecutive. This configuration remained constant for all trials within a day. Reward arms were baited with half a chocolate vermicelli strand, with secondary wells behind all arms filled to mask scent. Mice performed ten trials daily, aiming to find all four reward arms per trial.

Each test day began with an 8-minute ‘exploration’ phase allowing mice to learn reward locations, and ensuring the task was reliant on memory. This was followed by 2-minute holding box intervals for maze cleaning and well replenishment - which was repeated between all trials.

Following the initial exposure to the reward configuration, testing consisted of two behavioural sessions separated by a 45 minute rest recording. Each behavioural session comprised five trials separated by 2-minute re-setting procedures. Trials lasted 4 minutes or until all reward arms were visited, whichever occurred first. Mouse position was tracked via overhead camera with manual arm entrance recording. Rest recordings lasted 45 minutes with mice held in a cylindrical rest box on the centre platform for continued position tracking and remote monitoring.

### Electrophysiology Recordings and Spike Sorting

Each microdrive contained two 32-channel Omnetics connectors for Intan headstage attachment. Headstages amplified and transmitted neural signals to an Open Ephys acquisition board, with raw traces continuously reviewed and stored in NWB format^66^ sampled at 30kHz.

To monitor cells across behavioural sessions and rest recordings, entire daily recordings were combined. Initial clustering used Kilosort 2.0^67,68^ for template-based spike classification, followed by manual refinement in Phy^69^. Amplitude, waveform, and temporal autocorrelation were analysed to reassign spikes and correct over-clustering. Completed spikes were reassigned to trials in Matlab.

Given unknown AD effects on firing properties, broad classification criteria were applied. Cells were categorized as pyramidal or interneurons based on waveform width using histogram analysis of peak-to-trough durations. The pyramidal cell threshold was set at the minima between two clear peaks (400µs), with shorter durations classified as interneurons.

Putative pyramidal cells were assessed for spatial properties to determine place cell classification. Firing fields required ≥9 connected 2cm bins exceeding twice mean firing rate. Place cells required ≥1 firing field, peak firing rate 1-50Hz, and mean firing rate <5Hz (based on Tanni et al.^70^).

### Position Tracking

Headstages were fitted with two infrared LEDs tracked by an overhead night-vision camera. X,Y positions were recorded using the PosTracker plugin for Open Ephys (github.com/rhayman/PosTracker) at approximately 30Hz with some variability. Position was interpolated and matched to neural signals using nearest-neighbour timestamp searches.

Occasional LED detection failures or false light source detection produced position jumps. Post-hoc removal of jumps exceeding one standard deviation from mean position change was performed in Matlab, with interpolation replacing missing data points.

Running speed was calculated using Pythagoras’ theorem for position change rate, adjusted for camera pixels-per-meter calibration, and averaged to determine distance travelled in centimetres per second.

## Quantification and Statistical Analysis

### Ratemaps

Position data and spikes were filtered for running speeds >2cm/s. Position samples were binned into 2cm x 2cm spatial bins, with firing ratemaps calculated by dividing spike counts by dwell time per bin. Ratemaps were Gaussian smoothed (σ=4cm). Mean rate was calculated as the average firing rate across all spatial bins, while peak firing rate was the maximum bin firing rate.

### Task Performance Scores

Behavioral performance was evaluated using scores based on unique arm preference and reward arm preference. These scores accounted for the number of choices made during each trial by comparing observed data to shuffled distributions of error probabilities. Separate shuffled distributions were generated for both preference measures. Each score was calculated as the inverse of the summed probability of making the observed number of errors or fewer, given the number of arm entrances.

Statistical analysis used linear mixed-effects modelling to account for individual mouse variations and assess multiple variable contributions. For both performance scores (unique arm preference and reward arm preference), the following model was applied:

LMEmodel=performanceScore∼1+Trial∗Day+Trial∗Genotype+Day*Genotype+Trial:Day:Genotype+(1|Mouse)

### Spatial Information

Spatial information values quantified the spatial specificity of each cell’s activity, with broadly firing cells having lower values than cells with condensed firing fields. To avoid novelty effects, spatial information was calculated using neural data from the second session of each test day.

Spatial ratemaps followed the adaptive binning method of Skaggs et al.^71^; the firing rate in each bin was calculated by expanding a circle around the bin until the conditions of the following equation were met:

(1) $$N_{spikes\;}>\frac{\alpha }{N_{occ\;}^{2}r^{2}}$$

Where *N*_*occ*_ is the number of occupancy samples falling within the circle, *N*_*spikes*_ is the number of spikes occurring within the circle, *r* is the radius of the circle in bins and *α* is a scaling parameter, set at 200 in this instance. The value within each bin is therefore *Rs* · *N*_*spikes*_ / *N*_*occ*_ where *Rs* refers to the position sampling rate.

Spatial information values were then attained using the method described by Skaggs *et al*.^72^. The information in bits per second was calculated using the formula:

(2) $$I_{sec}=\lambda (x)\log_{2}\frac{\lambda (x)}{\lambda }p(x)dx$$

Where *x* is the spatial location and *p* (*x*) is the probability density of the mouse being in that location. *λ*(*x*) is the mean firing rate at position *x*. The overall mean firing rate of the cell is calculated as:

(3) $$\text{λ}=\sum_{x}^{□}\lambda (x)p(x)dx$$

The spatial information in bits per spike can then be calculated for each cell using the equation:

(4) $$I_{spike\;}=\frac{I_{sec\;}}{\lambda }$$

### Spatial Correlations

To assess place cell stability, we used spatial correlations to compare rate maps. For across-session stability, separate rate maps were generated for sessions one and two on each recording day for each place cell, then compared using bin-wise Pearson correlations excluding unvisited bins. Odd-even stability was assessed by generating separate rate maps for ‘odd minutes’ (1, 3, etc.) and ‘even minutes’ (2, 4, etc.), then correlating these similarly. To assess temporal dynamics, individual rate maps were created for every minute across both sessions, with all pairwise comparisons at different time lags performed using the same correlation method.

Stability changes across days, trials, and between genotypes were assessed using linear mixed-effects modeling. For both across-session and odd-even correlations, the following model was used:

LMEmodel=CorrelationValue∼1+Genotype*Date+(1|Mouse)

Temporal dynamics of within-session stability changes used:

LMEmodel=CorrelationValue∼1+Session*Genotype+TimeLag*Genotype+(1|Mouse)

### LFP Analysis

Local field potentials (LFPs) were sampled at 250 Hz and notch-filtered at 50 Hz (bandwidth = 1.4 Hz) using a zero-phase IIR filter to remove line noise. The filtered signal was segmented into 2 s windows with 50% overlap for spectral analysis. The LFP was then band-pass filtered within the theta (6–12 Hz) and delta (2–4 Hz) ranges, and the Hilbert transform was applied to extract the instantaneous amplitude and frequency. Slow-wave sleep was defined as periods of rest in which the theta–delta ratio (θ/δ) fell more than one standard deviation below the mean θ/δ ratio during session 1.

### Ripple Detection

LFPs sampled at 1200 Hz were used to identify ripples from the same electrodes implanted in CA1 as the main dataset. Ripples were defined as transient bursts of activity in the 150–250 Hz range where power exceeded four standard deviations above the mean during rest. Ripple envelopes were extended bidirectionally from the peak power until the signal returned to the mean.

### MUA Detection

Reactivation events were identified as epochs where multi-unit activity (MUA) of all place cells exceeded three standard deviations above mean rest recording activity. MUAs were detected throughout the 45 minute rest recordings and were not restricted to a specific sleep stage, in line with previous studies^73–75^. MUA levels were assessed using histograms of all cell spike activity in 10ms bins, smoothed with a Gaussian kernel (σ=15ms) and z-scored. Potential MUA events were identified as periods exceeding 3 standard deviations above the mean.

Activity peaks passing this threshold were identified, with MUA boundaries extended bidirectionally until firing returned to baseline. For inclusion, MUA events required >40ms duration and activity from minimum 5 cells or 10% of total cells (whichever was greater). Cell activity was defined as ≥1 action potential within the MUA duration. These criteria align with previous studies^8,47,76^.

### MUA Structure

To determine the structure of MUA content, we developed a score measuring the extent to which place cell co-firing differed from chance expectations assuming statistical independence. We defined the probability of the *i*^*th*^ cell being active in any given MUA as:

(5) $$P_{i}=\frac{n_{i}}{N}$$

Where *n*_*i*_ is the number of MUA events in which cell i is active and N is the total number of MUA events in that rest session.

Under statistical independence, the expected probability that any given cell pair (*i* & *j*) would co-activate in the same MUA events is the joint probability:

(6) $$P_{\exp\;ij}=P_{i}\times P_{j}$$

The observed probability of cells *i* and *j* co-activating within MUAs was calculated as:

(7) $$P_{obs\;ij}=\frac{n_{ij}}{N}$$

Where *n*_*ij*_ is the number of MUA events in which both *i* and *j* were active. To quantify departure from expected probabilities, we calculated a z-score and took the absolute value:

(8) $$Z=\frac{\left|P_{obs}j-P_{\exp ij}\right|}{\text{sqrt}\left(\left(P_{\exp ij}\times 1-P_{\exp ij}\right)/N\right)}$$

Where *N* is the total number of MUA events. Higher absolute Z-values indicate greater structure in MUA content, with values approaching zero suggesting random co-activation patterns (observed matches expected under independence).

For each cell, the MUA structure score was the average structure score of all MUAs to which it was recruited. The relationship between MUA structure and the z-transform of the across session ratemap correlation was assessed using the linear mixed effects model:

LMEmodel=z−ScoredSessionCorrelation∼1+MUAstructure+(1|Mouse:Day)

### Replay Decoding

Decoding of MUA content followed Ólafsdóttir & Barry^8^ consisting of three steps: (1) identifying the maze arm best represented by neural data, (2) generating a posterior-probability matrix for the selected arm, and (3) fitting and validating a best-fit trajectory. Replay events were classified as forward (>2cm/s), reverse (<-2cm/s) or stationary based on the direction of the fitted trajectory. Directionality was quantified separately for periods of awake immobility (‘quiescence’) and slow-wave sleep (SWS) (Figure S4), however all events recorded in the rest session, regardless of brain state or direction, were included in the main analyses.

#### Maze arm selection

Ratemaps for each arm were split into inbound and outbound trajectories, then combined into single long ratemaps. All place cell ratemaps were stacked to produce a matrix. Using a Bayesian approach, expected spike counts for each cell in each spatial bin were calculated based on combined ratemaps. Neural data was binned into 10ms intervals, with observed spikes compared to expected spikes across spatial bins using a Poisson noise model. This produced a posterior probability matrix (N_spatial bins_ x N_time bins_) for each MUA event, normalized so each time bin column summed to one. The arm and direction with the highest total probability across all time and spatial bins was selected for subsequent analysis.

#### Single-arm posterior-probability

The posterior-probability matrix was recalculated using only ratemaps from the selected arm and direction, with probabilities renormalized across the track.

#### Trajectory fitting

Linear fit lines (width 10 bins, 20cm) were exhaustively applied sampling all gradients (V) between ±50m/s in 0.5m/s increments and all possible intercepts (c). The slope accounting for the highest probability matrix proportion was deemed best fit. Goodness of fit R(V,c) was calculated as:

(9) $$R(V,c)=\frac{1}{n}\sum_{t=0}^{n-1}P(|\times (t)-(V\cdot t\cdot T+c)|\leq d)$$

Where *t* indexes the time bins of width *T*, and *d* represents the 20cm band width. The trajectory was required to have a length of at least a quarter of the duration of the MUA to be accepted.

#### Significance testing

Two shuffle procedures validated trajectory significance. First, cell ID shuffles reassigned spikes to other active cells’ ratemaps, repeating all analysis steps 100 times. MUAs exceeding 95% of shuffled goodness-of-fit values proceeded to secondary testing. Second, circular column shifts of the posterior-probability matrix were applied (maximum shift = matrix height), with trajectory fitting repeated 100 times. MUAs exceeding 95% of shuffled fits were accepted as significant replay trajectories, with their goodness-of-fit termed ‘quality of fit’.

### Histology and Plaque Measurement

Brains were sectioned using either a freezing cryostat or vibrotome to yield 50µm horizontal slices. Five sections spanning the hippocampus were taken from 7 of the WT and 5 of the NL-G-F mice. Sections were dual-stained with thioflavinS (1:100 dH2O) for quantification of plaque volume in the hippocampus and DAPI. Slides were re-hydrated in dH_2_O then submerged in the thioflavinS staining solution for 8 minutes. They were then dehydrated in increasing concentrations of IMS and mounted using a Vectashield® mounting solution containing DAPI for nuclei staining.

ThioflavinS slides were imaged at 10x magnification on the epifluorescence microscope settings in the Axioscan slide scanner. Images were captured in the 330-375 excitation range and 430-470 emission range for DAPI, and the 453-485 excitation and 504-546 emission range for ThioflavinS, which is a green fluorescent stain. DAPI images were used to identify the bilateral hippocampus as the region of interest (ROI) for calculation of plaque volume. The thiolflavinS image was thresholded using an Otsu filter and binarized, such that pixels passing the threshold were seen in white. The bilateral hippocampal pixel area was measured, and plaque volume expressed as the proportion of pixels exceeding the Otsu threshold. This was repeated across five brain sections per mouse, to give a single averaged plaque volume per mouse.

## KEY RESOURCES TABLE

**Table:** 

REAGENT or RESOURCE	SOURCE	IDENTIFIER
Antibodies		
THIOFLAVINE S	Merck Life Science UK	T1892-25G
Vectashield with DAPI	2bScientific	H-1800-10
Experimental models: Organisms/strains		
Mice: App^tm3.1Tcs^/App^tm3.1Tcs^	Saito et al.^41^	NA
Mice: 129S6-Chat^tm2(cre)Lowl^/J	The Jackson Laboratory	Strain #006410
Software and algorithms		
MATLAB	Mathworks	2023a
KILOSORT	Pachitariu et al.^67^	github.com/MouseLand/Kilosort
Phy	Rossant et al.^69^	github.com/cortex-lab/phy
Open Ephys	Siegle et al.^66^	open-ephys.org
Custom Matlab code	This paper	DOI: 10.5281/zenodo.17976972
**Deposited data**		
Figshare - Supporting data for: Disrupted hippocampal replay is associated with reduced offline map stabilisation in an Alzheimer’s mouse model	This paper	https://doi.org/10.5522/04/30902972
**Other**		
Open EPhys Acquisition Board	Open Ephys Organisation	https://open-ephys.org/acquisition-system/eux9baf6a5s8tid06hk1mw5aafjdz1
Intan Standard SPI interface cable	Intan Technologies	RHD2000 3-ft (1.8 m),
Intan amplifier/accelerometer board with 64 unipolar inputs	Intan Technologies	RHD2164
32 channel connector, Omnetics	Genalog	A79621-001
12.7 μm HM-L coated Stablohm 650	California Fine Wire Company	100187
Single-screw mouse microdrive	Axona	Product code: MDMR-01M1,
NanoZ plating equipment	Multichannel Systems	nanoZ

## Supplementary Material

Supplementary Information

## Data Availability

All data have been deposited at figshare (UCL Research Data Repository) and are publicly available as of the date of publication. DOIs are listed in the key resources table. All original code has been deposited on Zenodo and is publicly available as of the date of publication. DOIs are listed in the key resources table. Any additional information required to reanalyse the data reported in this paper is available from the lead contact upon request.
