# Supplementary material for: Disrupted hippocampal replay is associated with reduced offline map stabilisation in an Alzheimer’s mouse model
Source: Curr Biol. Author manuscript; Available in PMC 2026 Oct 8. (PMC7619514; doi:10.1016/j.cub.2025.12.061)
Supplement: Supplementary file 1 [file EMS219676-supplement-Supplementary_Information.pdf]

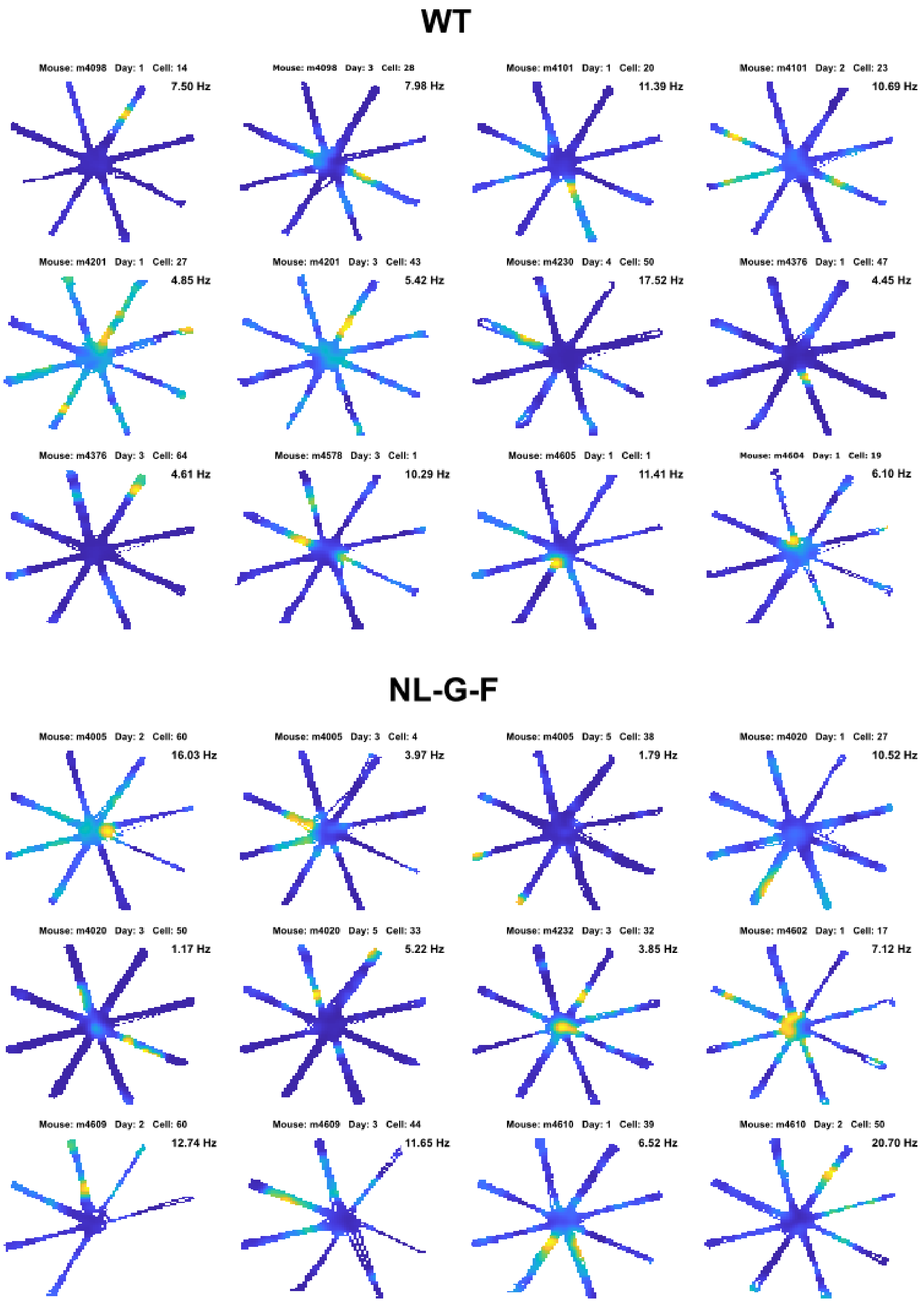

Figure S1. **Example ratemaps of place cells recorded on the radial-arm maze. Related to Figure 3.** Mouse ID, recording day and cell ID are given above each ratemap. Peak firing rate is provided at the top-right of each plot. Colours are scaled separately, from zero to the peak firing rate, for each cell. Unvisited bins are left white.

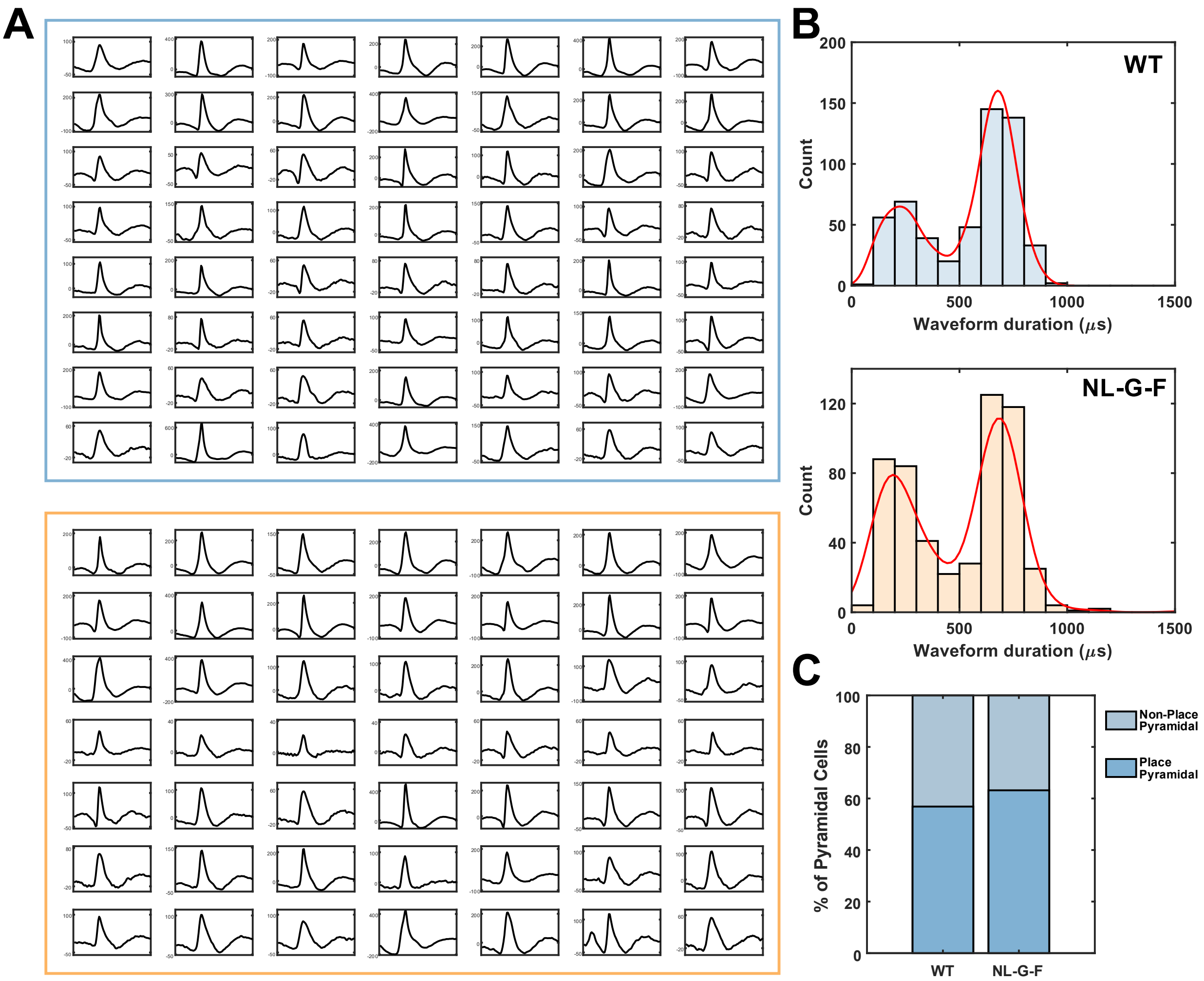

Figure S2. Waveform characteristics. Related to Figure 3. A. Example waveforms of place cells recorded from WT (blue box) and NL-G-F (orange box) mice. Each row contains example cells recorded from a single mouse. Each waveform is shown as a 1 ms window, averaged from 100 samples. **B.** Histograms of all recorded waveform durations on the first day of recording in WT (top) and NL-G-F (bottom) mice with KDE overlaid (Red). The minimum waveform duration, measured peak to trough, for classification as a pyramidal cell was 400μs. The wave duration of pyramidal cells was not different between the two genotypes (M_{WT}=662.0, $M_{NL\text{-}G\text{-}F}$=674.5, t(710)=-1.76, p=0.12). **C.** The proportion of pyramidal cells which were categorised as place cells was similar across genotypes (M^{WT}=56.8, $M^{NL\text{-}G\text{-}F}$=63.1, t(13)=-0.79, p=0.44).

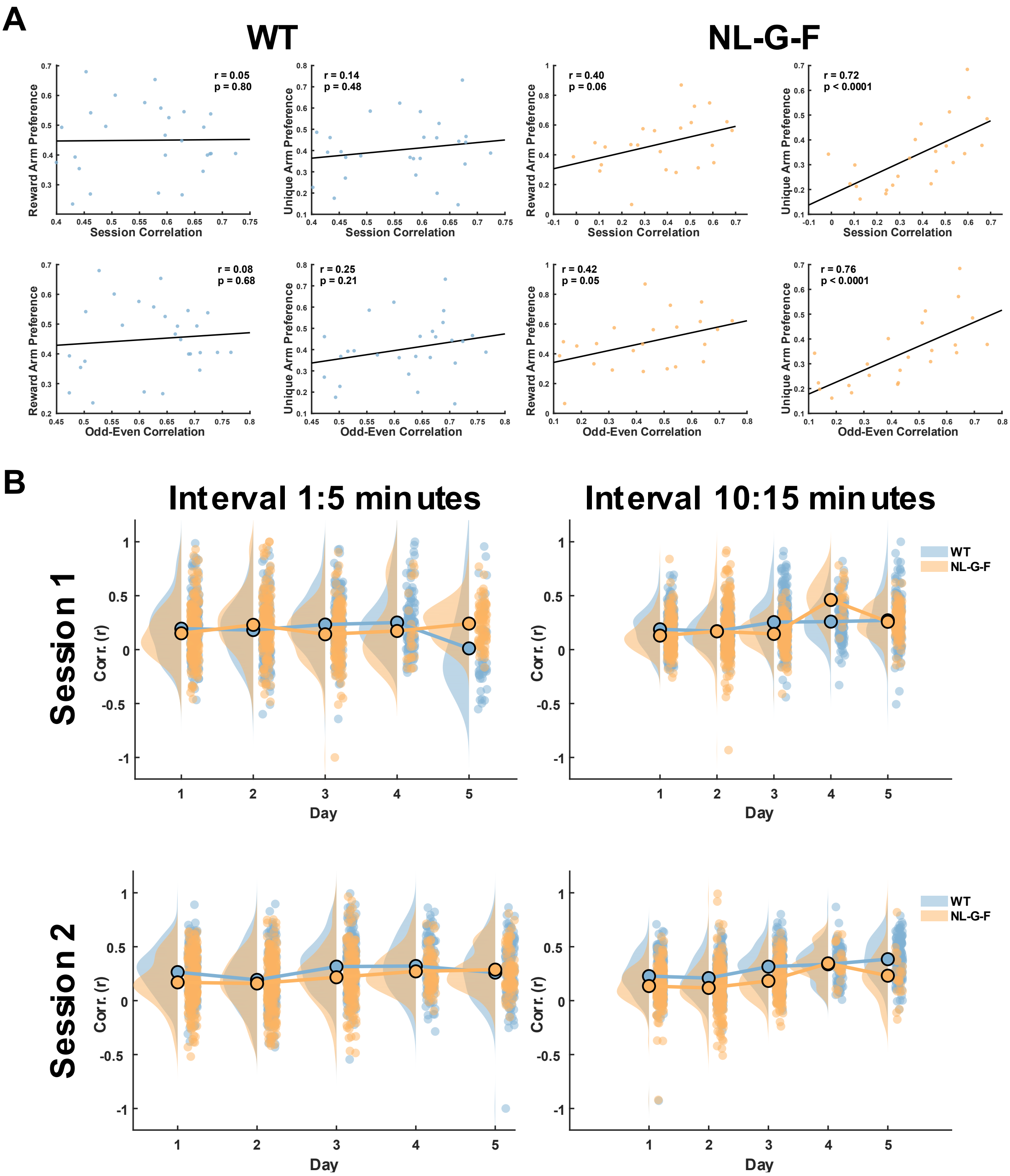

Figure S3. Additional place cell stability measures. Related to Figures 4,5. A. Correlation between stability measures and memory performance scores. Significant positive correlations exist for NL-G-F mice between both measures of stability (Odd-Even Minute correlations and Across Session correlations) and unique arm preference scores. WT mice show less variability in stability scores, though a non-significant positive trend remains. Similar trends are seen for NL-G-F mice between stability and reward arm preference scores. The line represents Spearman correlation. Each dot represents a recording day for a single mouse; n_{WT}=27, $n_{NL\text{-}G\text{-}F}$=23. **B.** Place cell stability over increasing time intervals across days. Individual one-minute ratemaps were compared over increasing time intervals within sessions. For visualisation, correlations were grouped into short (1-5 minutes) and long

(10-15 minutes) interval comparisons. The LME model from Figure 3 was extended to include day as a fixed-effect (LME model: *correlation value ~ 1 + day*genotype + session*genotype + time lag*genotype + (1 | mouse)*). There were significant main effects of day (Estimate: 0.02, t(295980)=20.86, p<0.0001), session (Estimate: 0.04, t(295980)=16.67, p<0.0001), time lag between ratemaps (Estimate: -0.004, t(295980)=-14.41, p<0.0001), and genotype (Estimate: -0.08, t(295980)=-2.78, p=0.005). There were also interaction effects of day and genotype (Estimate:-0.004, t(295980)=-2.32, p= 0.02), session and genotype (Estimate:-0.02, t(295980)=-5.87, p<0.0001) and time lag and genotype (Estimate:0.002, t(295980)=5.23, p<0.0001). Only overlapping spatial bins were analysed (n = 305,688 WT and 228,781 NL-G-F ratemap pairs).

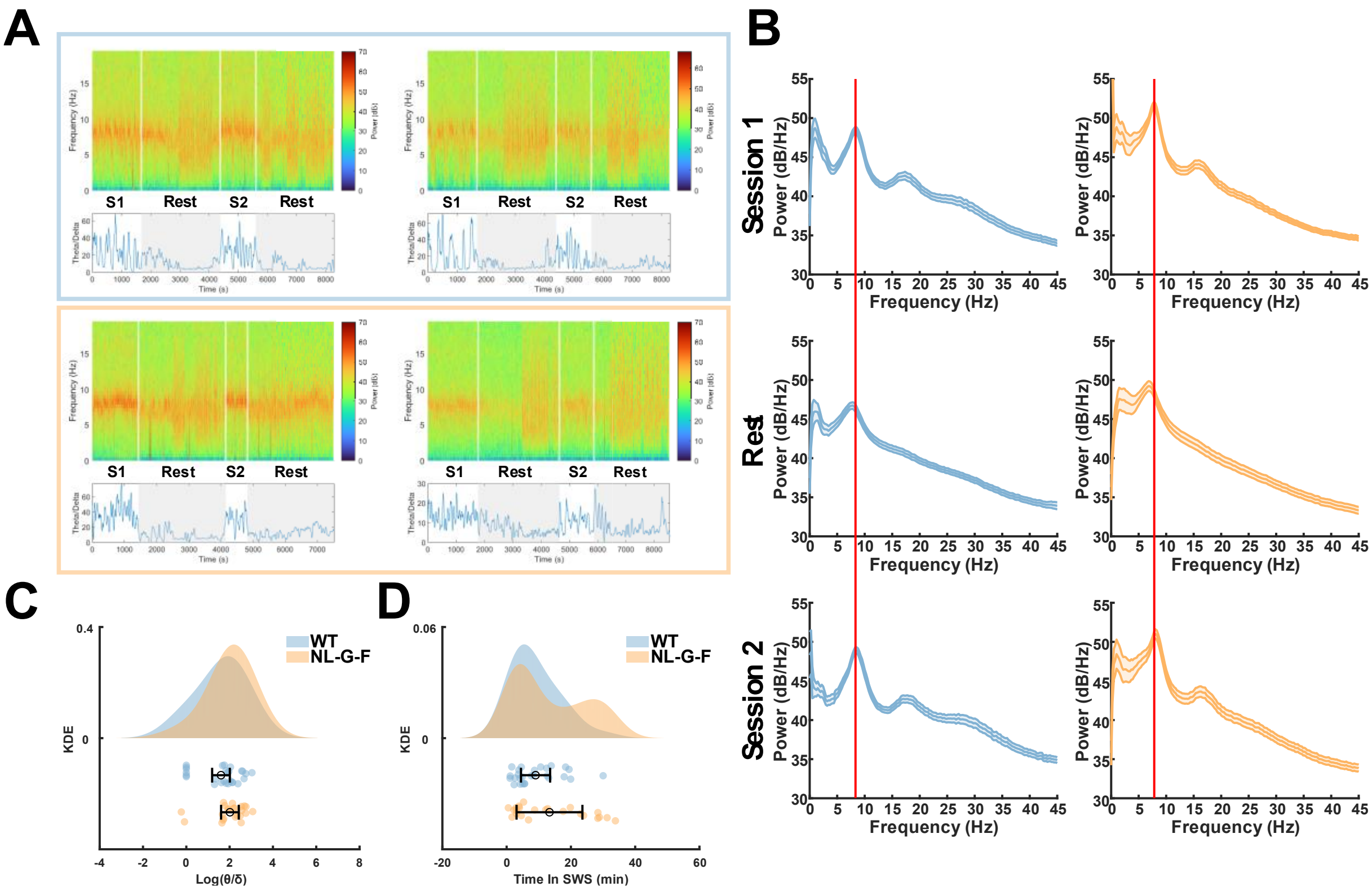

Figure S4. Brain states in rest periods are similar in WT and NL-G-F mice. Related to Figure 6. A. Example spectrograms of full day recordings from WT (blue box) and NL-G-F (orange box) mice. White lines separate out the different recordings - session 1, rest 1, session 2, rest 2 (n.b. Only the first rest recording was included in all analyses). The theta-delta ratio is plotted below, grey boxes represent rest recordings. **B.** Power spectral density plots for WT (left) and NL-G-F (right) mice across session 1, rest and session 2 recordings. Lines represent the average value across mice and days, and the shaded area represents the SEM. Plots are aligned vertically, and red lines represent the position of the peak frequency in session 1. **C.** There was no difference in the overall logged theta-delta ratio in rest 1 between the genotypes (Two-sample t-test, t(482)=-1.64 0.086, p=0.110.93, M_{WT}=1.62.23, $M_{NL\text{-}G\text{-}F}$=2.012.21). **D.** The total time in slow-wave-sleep (periods of the rest recording with a θ/δ ratio one standard deviation below the mean waking θ/δ ratio) was not different between the two genotype groups. (Two-sample t-test, t(482)=-1.90.88, p=0.0639, M_{WT}=13.3524, $M_{NL\text{-}G\text{-}F}$=8.0611.63). n_{WT}=27, $n_{NL\text{-}G\text{-}F}$=23.

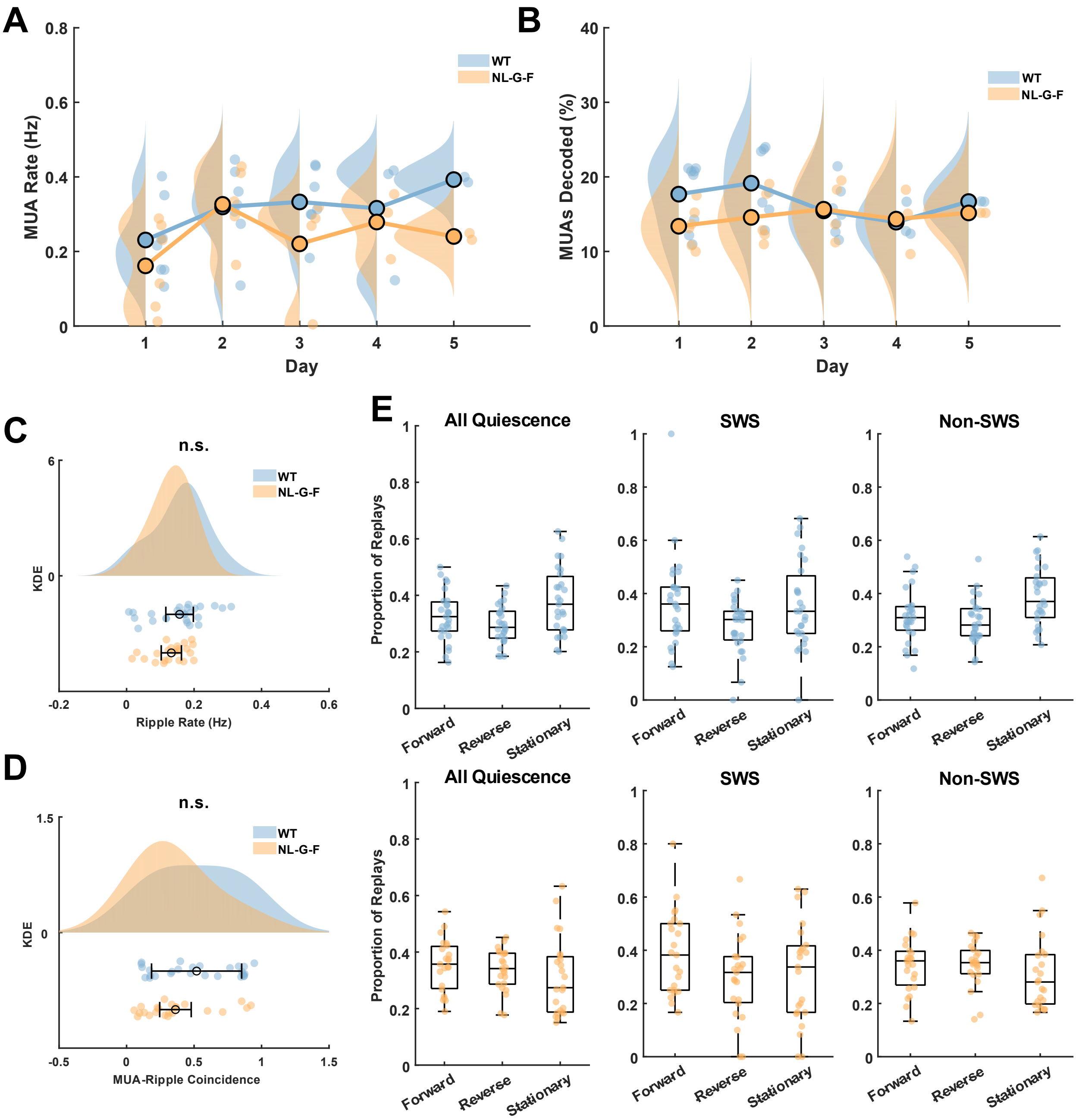

Figure S5. Additional analyses of reactivation and replay decoding. Related to Figure 6. A. The MUA rate was consistently similar between genotypes across days. LME model: *muaRate ~ 1 + Day*Genotype + (1 | Mouse).* There was no main effect of genotype (Estimate: -0.036, t(43)=-0.67, p=0.50), Day (Estimate: 0.002, t(43)=-0.12, p=0.91) or interaction of the two (Estimate: 0.029, t(43)=-0.02, p=0.13). **B.** The percentage of MUAs containing an replay trajectory passing the criteria for inclusion was decreased in NL-G-F mice (Estimate: -5.98, t(43)=-2.58, p=0.013) however there was no main effect of day (Estimate:-0.84, t(43)=-1.53, p=0.13) or interaction (Estimate: 1.44, t(43)=- 1.81, p=0.08). LME model: *Percentage Decoded ~ 1 + Day*Genotype + (1 | Mouse).* Scatter plots show a value per mouse. **C.** The rate of ripple detection in the rest recording between sessions was similar in WT and NL-G-F mice (M_{WT}=0.15, $M_{NL\text{-}G\text{-}F}$=0.13, t(48)= 1.28, p=0.21). **D.** The coincidence of ripples and MUAs was also not significantly different across the two genotypes (M_{WT}=0.51, $M_{NL\text{-}G\text{-}F}$=0.36, t(48)=1.94, p=0.06). Scatter plots show individual rest recordings (n_{WT}=27, $n_{NL\text{-}G\text{-}F}$=23 mouse-days). **E.**

The brain state did not impact the proportion of forward, reverse and stationary replay events in rest. Boxplots show median ± IQR of the proportion of forward, reverse, and stationary replay events across mice for each brain state (SWS, non-SWS, rest) and genotype (WT: blue, top. NL-G-F: orange, bottom). Scatter plots show individual mouse-days (n_{WT}=27, $n_{NL\text{-}G\text{-}F}$=23). LME model: *replay direction ~ 1 + Genotype*Brain state + (1| Mouse:Day)*. **Forward Replay:** There was no main effect of Genotype (Estimate= 0.03, t(144)=0.78, p=0.43) or brain state (SWS: Estimate=0.04, t(144)=1.9, p=0.05; non-SWS: Estimate=-0.01, t(144)=-0.48, p=0.63) and no interaction effects (genotype x SWS: Estimate = -0.006, t(144)=-0.19, p=0.84; genotype x non-SWS: Estimate=0.0008, t(144)=0.02, p=0.98. **Reverse Replay:** There was no main effect of Genotype (Estimate= 0.04, t(144)=0.03, p=0.14) or brain state (SWS: Estimate=-0.02, t(144)=-0.88, p=0.38; non-SWS: Estimate=0.001, t(144)=0.04, p=0.96) and no interaction effects (genotype x SWS: Estimate = -0.02, t(144)=-0.-0.53, p=0.59; genotype x non-SWS: Estimate=0.009, t(144)=0.29, p=0.77). **Stationary Replay:** There was no main effect of Genotype (Estimate= -0.07, t(144)=-1.70, p=0.09) or brain state (SWS: Estimate=-0.02, t(144)=-1.39, p=0.16; non-SWS: Estimate=0.009, t(144)=0.57, p=0.56) and no interaction effects (genotype x SWS: Estimate = 0.02, t(144)=0.92, p=0.35; genotype x non-SWS: Estimate=-0.009, t(144)=-0.40, p=0.69).

WT

Mouse	Day	Age	Place Cells
m0168	1	12	-
m0168	2	12	-
m0168	3	12	-
m0168	4	12	-
m0168	5	12	-
m4098	1	16	65
m4098	2	16	52
m4098	3	16	51
m4098	4	16	41
m4098	5	16	57
m4101	1	16	47
m4101	2	16	47
m4101	3	16	44
m4101	4	16	-
m4101	5	16	-
m4201	1	8	39
m4201	2	8	58
m4201	3	8	43
m4201	4	8	-
m4230	1	12	23
m4230	2	12	21
m4230	3	12	40
m4230	4	12	58
m4230	5	12	83
m4376	1	17	20
m4376	2	17	31
m4376	3	17	32
m4376	4	17	14
m4578	1	11	14
m4578	2	11	23
m4578	3	11	15
m4604	1	9	10
m4604	2	9	9
m4604	3	9	14
m4605	1	9	14
m4605	2	9	-
m4605	3	9	-

NL-G-F

Mouse	Day	Age	Place Cells
m0166	1	12	-
m0166	2	12	-
m0166	3	12	-
m0166	4	12	-
m0166	5	12	-
m4005	1	15	20
m4005	2	15	23
m4005	3	15	27
m4005	4	15	24
m4005	5	15	30
m4020	1	14	59
m4020	2	14	61
m4020	3	14	52
m4020	4	14	51
m4020	5	14	54
m4202	1	7	9
m4202	2	7	19
m4202	3	7	15
m4232	1	12	51
m4232	2	12	56
m4232	3	12	60
m4602	1	10	7
m4602	2	10	18
m4602	3	10	-
m4602	4	10	-
m4602	5	10	-
m4609	1	11	17
m4609	2	11	15
m4609	3	11	25
m4610	1	11	59
m4610	2	11	54
m4610	3	11	-

Table S1. Number of place cells recorded across mice and days. Related to Figure 3. Mouse ID, days into the experiment, age (months) and number of place cells for each experimental day. Days where no place cell number is recorded were included in behavioural analyses only.

Equation	Figure	Estimates	p Value
*Reward Arm Performance Score ~ 1 + Trial*Day + Trial*Genotype + Day*Genotype + Trial:Day:Genotype + (1 | Mouse)*	2A-B	Trial: 0.026	**<0.01**
		Genotype: 0.188	0.062
		Day: 0.034	0.102
		Trial x Day: -0.004	0.281
		Trial x Genotype: -0.025	0.084
		Day x Genotype: -0.052	0.09
*Unique Arm Performance Score ~ 1 + Trial*Day + Trial*Genotype + Day*Genotype + Trial:Day:Genotype + (1 | Mouse)*	2C-D	Trial: -0.024	**<0.05**
		Genotype: -0.243	**<0.05**
		Day: -0.060	**<0.01**
		Trial x Day: 0.01	**<0.01**
		Trial x Genotype: 0.017	0.24
		Day x Genotype: 0.070	**<0.05**
*Normalised Firing Rate ~ 1 + Genotype*Running Speed + (1 | Mouse) + (1 | Day) .*	3C	Genotype: 0.08	**<0.01**
		Speed: 0.006	**<0.0001**
		Genotype x Speed: 0.007	0.32
*Across Session Correlation ~ 1 + Genotype*Runs through Field + Genotype*Time in Field + (1 | Mouse:Date)*	3F-G	Genotype: –0.14	**<0.01**
		Runs through Field: -0.002	**<0.0001**
		Time in Field: 0.004	**<0.0001**
		Genotype x Runs: 0.0003	0.25
		Genotype x Time: -0.0008	0.055
*Odd-Even Correlation value ~ 1 + Genotype*Day + (1 | Mouse)*	4A	Genotype: -0.23	**<0.001**
		Day: 0.02	**<0.01**
		Day x Genotype: 0.007	0.45
*Across Session Correlation value ~ 1 + Genotype*Day + (1 | Mouse)*	4B	Genotype: -0.28	**<0.0001**
		Day: 0.02	**<0.001**
		Day x Genotype: 0.02	**<0.05**
*One-Min Ratemaps Correlation value ~ 1 + Session*Genotype + Time Lag*Genotype + (1 | mouse)*	5A-B	Genotype: -0.098	**0.001**
		Time Lag: -0.005	**<0.0001**
		Session: 0.04	**<0.0001**
		Session x Genotype: -0.03	**<0.0001**
		Time Lag x Genotype: 0.003	**<0.0001**
z-Transformed Across Session Correlation ~ 1 + MUA inclusion + (1 | Mouse:Day)	6G	MUA Inclusion: 0.003	**<0.0001**
z-Transformed Across Session Correlation ~ 1 + MUA Structure Score + (1 | Mouse:Day)	6H	MUA Structure: 0.101	**<0.0001**
*One-Min Ratemaps Correlation value ~ 1 + Day*Genotype + Session*Genotype + Tme Lag*Genotype + (1 | Mouse).*	S3	Genotype:-0.08	**<0.01**
		Day: 0.02	**<0.0001**
		Time Lag: -0.004	**<0.0001**
		Session: 0.04	**<0.0001**
		Session x Genotype: -0.02	**<0.0001**
		Time Lag x Genotype: 0.002	**<0.0001**
		Day x Genotype: -0.004	0.05
*MUA Rate ~ 1 + Day*Genotype + (1 | Mouse)*	S5	Genotype: -0.03	0.5
		Day: 0.002	0.91
		Day x Genotype: 0.029	0.13
*MUA Percentage Decoded ~ 1 + Day*Genotype + (1 | Mouse)*	S5	Genotype: -5.98	**<0.05**
		Day: -0.84	0.13
		Day x Genotype: 1.45	0.08

Table S2. Summary of linear mixed effects models' results. **Related to Figures 2,3,4,5,6.** All 'Genotype' effects are given as differences in NL-G-F data from WT baseline. In all cases, individual variability across mice was controlled for.
